# QRICH1 Disrupts Endoplasmic Reticulum Homeostasis and Amplifies NF‐κB Signaling in Periodontal Ligament Stem Cells to Exacerbate Diabetic Periodontitis

**DOI:** 10.1002/advs.76650

**Published:** 2026-07-20

**Authors:** Han Li, Xiaoyu Yang, He Wang, Yunchun Kuang, Yiyao Hu, Houxuan Li, Shuhong Li, Deping Zeng, Jie Li, Jinlin Song

**Affiliations:** ^1^ College of Stomatology Chongqing Medical University Chongqing People's Republic of China; ^2^ Chongqing Key Laboratory For Oral Diseases Chongqing People's Republic of China; ^3^ Chongqing Municipal Key Laboratory of Oral Biomedical Engineering of Higher Education Chongqing People's Republic of China; ^4^ Chongqing Municipal Health Commission Key Laboratory of Oral Biomedical Engineering Chongqing People's Republic of China; ^5^ State Key Laboratory of Ultrasound in Medicine and Engineering Chongqing Medical University Chongqing People's Republic of China

**Keywords:** apoptosis, diabetic periodontitis, endoplasmic reticulum stress, glutamine‐rich protein 1, inflammation, periodontal ligament stem cells, protein homeostasis

## Abstract

Functional impairment of periodontal ligament stem cells (PDLSCs) drives tissue destruction in diabetic periodontitis. While chronic, unresolved endoplasmic reticulum (ER) stress is known to compromise PDLSC stemness, the precise underlying mechanisms remain elusive. Recent studies identify glutamine‐rich protein 1 (QRICH1) as a crucial regulator governing cell entry into terminal or adaptive unfolded protein response (UPR). Herein, analysis of single‐cell transcriptomic datasets reveals that QRICH1 modulates fibroblast and osteoblast functions. QRICH1 is markedly elevated in human diabetic periodontal tissues and damaged PDLSCs, with its expression regulated by UPR. Transcriptomic profiling indicates enrichment in protein oligomerization, calcium homeostasis, and NF‐κB signaling in PDLSCs under high‐glucose and inflammatory conditions. Functional assays demonstrate that QRICH1 drives PDLSC dysfunction by sustaining global protein synthesis, amplifying NF‐κB activation through interaction with p65, and enhancing UPR‐mediated apoptosis. Moreover, transcriptomic analysis following QRICH1 depletion confirms the downregulation of biosynthetic and stress‐response pathways, corroborating its role in regulating protein homeostasis. In addition, QRICH1 knockdown effectively restores cellular function in vitro. Adeno‐associated virus (AAV)‐mediated *Qrich1* knockdown mitigates alveolar bone loss while suppressing ER stress and NF‐κB activation. Collectively, these findings reveal that QRICH1 drives PDLSC dysfunction by enhancing NF‐κB signaling and UPR‐mediated apoptosis, highlighting its therapeutic potential in diabetic periodontitis.

## Graphical abstract

QRICH1 has been established as a key factor contributing to impaired osteogenic potential and accelerated apoptosis of PDLSCs in diabetic periodontitis. QRICH1 not only significantly enhances UPR‐associated apoptotic signaling but also amplifies NF‐κB‐mediated inflammatory responses. Its inhibition restores osteogenic capacity and reduces alveolar bone loss, offering a novel perspective for repairing metabolic stress‐related tissue damage.

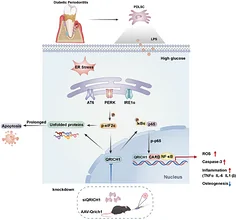

## 1 Introduction

Diabetes is a metabolic disorder prevalent worldwide [1], with periodontitis now recognized as its sixth complication [2, 3]. Current evidence suggests that the incidence of moderate to severe periodontitis among patients with type 2 diabetes mellitus (T2DM) is significantly higher than in healthy individuals [4, 5]. Due to the loss of periodontal attachment and severe bone destruction, along with suboptimal treatment outcomes, significant clinical challenges arise [6, 7]. Human periodontal ligament stem cells (hPDLSCs) possess self‐renewal and osteogenic differentiation capabilities, which are essential for periodontal immunoregulation and maintaining tissue integrity [8, 9]. However, the systemic inflammation, elevated oxidative stress, and impaired immunomodulatory responses associated with diabetes can compromise these cells [10, 11, 12]. These factors may result in weakened osteogenesis, expanded inflammation, and increased apoptosis of hPDLSCs, representing a core event in accelerated periodontal tissue destruction. Nevertheless, the underlying molecular mechanisms driving hPDLSC dysfunction in a high‐glucose inflammatory microenvironment remain incompletely elucidated.

Given that the endoplasmic reticulum (ER) is the main site for protein synthesis and post‐translational manipulation [13], its homeostasis is vital for maintaining the functional integrity of PDLSCs [14]. Under inflammatory and hyperglycemic conditions, excessive protein synthesis demands induce ER stress and protein misfolding/aggregation, which activates the unfolded protein response (UPR) [15, 16]. Current research indicates that ER stress plays a pivotal role in the progression of periodontitis [17]. Chronic periodontitis contributes to prolonged ER stress and sustained UPR, resulting in impaired osteogenic differentiation of PDLSCs [18, 19, 20]. Diabetes causes chronic hyperglycemia and hyperlipidemia, which also disrupt ER function [21, 22]. However, the restoration of ER homeostasis can significantly enhance the function of mesenchymal stem cells (MSCs) in a hyperglycemic microenvironment and improve the osteogenic potential of PDLSCs [23, 24]. Notably, persistent unresolved ER stress in diabetic periodontitis leads to the failure of ER adaptive regulation, driving cells toward terminal UPR and apoptosis, further disrupting periodontal tissue homeostasis [25]. Research suggests that under unmitigated ER stress, IRE1 and ATF6 signaling diminish, whereas activation of the protein kinase RNA–like endoplasmic reticulum kinase (PERK) remains largely sustained, resulting in UPR‐mediated cell death [26]. Activated PERK phosphorylates the α subunit of eukaryotic initiation factor 2 (eIF2α) and promotes subsequent downstream translational and transcriptional signaling [27]. Enhanced PERK/ATF4 signaling pathway leads to an imbalanced differentiation (adipogenic and osteogenic) of MSCs [28]. Inhibiting the PERK‐eIF2α pathway can modulate translation, thereby attenuating its effects on cells [29]. Furthermore, downregulation of the PERK axis significantly reduced intracellular calcium levels, oxidative stress, and apoptosis in inflammatory PDLCs [30]. However, how PERK signaling modulates hPDLSCs function in diabetic periodontitis remains to be investigated.

A recent study revealed that glutamine‐rich protein 1 (QRICH1) promotes proteotoxicity mediated by the UPR‐PERK axis and facilitates terminal apoptosis [31]. Previous studies have confirmed that QRICH1 is expressed in various tissues and organs and participates in inflammation, apoptosis, and immunoregulation through transcriptional regulation and protein interactions [31, 32, 33]. In addition, depletion of QRICH1 suppresses apoptosis and inflammation during hypertrophy [34]. QRICH1 overexpression leads to increased expression of ER stress‐related proteins and neuronal death [35]. Research also indicates that QRICH1 exhibits stronger signaling in the jawbone and limbs, and its mutations can affect skeletal growth and development [36]. Notably, QRICH1 contains a caspase activation recruitment domain (CARD) [37], and studies have indicated that CARD family proteins are crucial for the nuclear factor‐κB (NF‐κB) pathway activation [38, 39, 40]. It is well established that NF‐κB signaling participates in regulating various physiological and pathological processes, especially immune response and inflammation [41]. The critical role of NF‐κB in both periodontitis and diabetes has been established by numerous studies. In this respect, AGEs can increase the expression of inflammatory cytokines in oral epithelial cells via NF‐κB [42], and activate the NLRP3 inflammasome to exacerbate inflammatory responses in hPDLSCs [43]. Meanwhile, high‐glucose treatment increases NF‐κB nuclear localization and transcriptional activity in PDLSCs; inhibiting NF‐κB can rescue periodontal inflammation and bone destruction in diabetic mice [44]. In view of QRICH1 being a crucial component of the UPR‐PERK axis, we speculate that the upregulated QRICH1 expression in diabetic periodontitis potentially increases protein toxicity, induces hPDLSCs apoptosis, and exacerbates alveolar bone resorption and periodontal attachment loss via the NF‐κB pathway. However, the underlying mechanisms between QRICH1 and NF‐κB signaling remain unexplored.

In this study, using a high‐glucose inflammatory microenvironment, we show that QRICH1 impairs osteodifferentiation and accelerates apoptosis of hPDLSCs in vitro. Knockdown of *Qrich1* in diabetic periodontitis mice effectively reduces alveolar bone loss. Mechanistically, QRICH1 not only significantly enhances UPR‐associated apoptotic signaling but also amplifies NF‐κB‐mediated inflammatory responses. Together, our study unveils the pivotal role of QRICH1 in diabetic periodontitis and further elucidates the underlying regulatory mechanism, offering a novel perspective on the repair of periodontal tissue impairment.

## 2 Results

### 2.1 QRICH1 is Upregulated in Diabetic Periodontitis and Associated With ER Stress Activation

We first conducted integrated analyses of single‐cell RNA transcriptomic datasets related to diabetes and periodontitis. The GSE188217 dataset was used to explore cellular heterogeneity between WT and db/db mouse periodontal tissues. Following quality control and unsupervised clustering, we identified 13 transcriptionally distinct clusters (Figure S1a). Based on a previous study [45], these clusters were further subdivided into fibroblasts, epithelial cells, immune cells, and endothelial cells according to canonical lineage markers (Figure S1b,c). Among these, fibroblasts represented a major stromal population exhibiting clear transcriptional differences (Figure S1d). Specifically, the expression of QRICH1, a transcriptional regulator associated with stress responses, was markedly increased in db/db fibroblasts compared with WT counterparts, as evidenced by both violin plot analysis and UMAP feature visualization (Figure S1e,f). Furthermore, analysis of the GSE280908 dataset revealed an upward trend in QRICH1 expression in osteoblasts of T2DM‐associated periodontitis (T2DM+PD) mice compared to the periodontitis‐only (PD) group (Figure S1g–i). These findings collectively suggest that QRICH1 upregulation may contribute to diabetes‐associated alterations in fibroblast activity and matrix remodeling, as well as influence osteoblast function.

To investigate the expression of QRICH1, we collected periodontal ligament (PDL) tissues and cells from healthy individuals and periodontitis patients (with or without diabetes). Western blot analysis revealed that QRICH1 was markedly upregulated in periodontal ligament tissues and cells from patients with diabetic periodontitis (Figure 1a–d). This pathological environment elevated levels of crucial ER stress‐related proteins (p‐PERK, GRP78, CHOP) and pro‐apoptotic markers (cleaved caspase‐3, BAX) (Figure 1a–d). Concurrently, T2DM and periodontitis promoted the expression of *QRICH1* and ER stress‐related genes (*GRP78*, *PERK*, *IRE1α*, *ATF6*, *CHOP*) in hPDLSCs (Figure 1e). Transmission electron microscopy (TEM) assay displayed markedly swollen and dilated ER in hPDLSCs from the periodontitis and diabetic periodontitis groups, indicating impaired ER function (Figure 1f).

**FIGURE 1:**
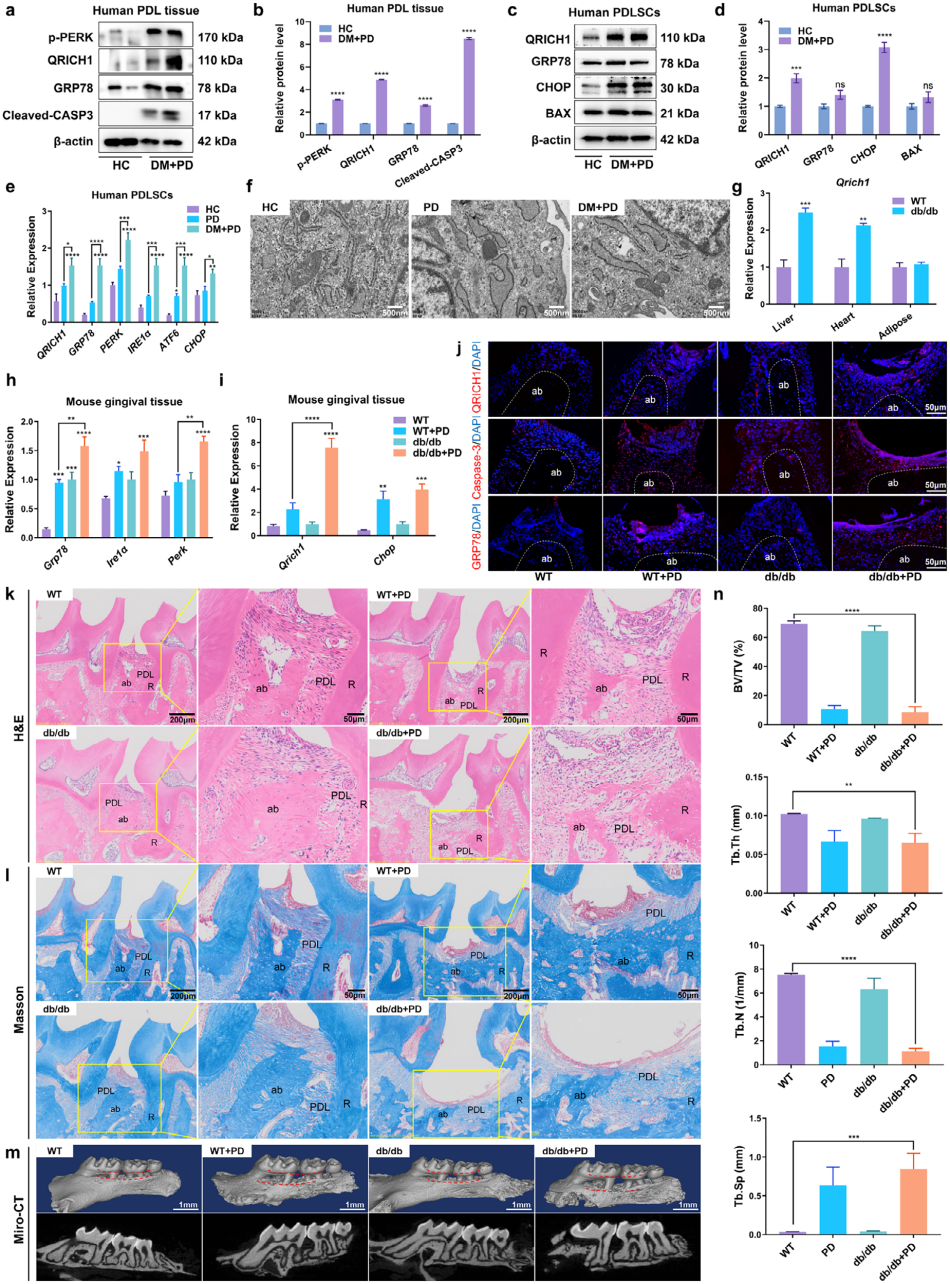
QRICH1 is upregulated in diabetic periodontitis and associated with ER stress activation. (a–d) Western blot analysis of QRICH1, ER stress markers (GRP78, p‐PERK, CHOP), and apoptosis markers (cleaved caspase‐3, BAX) in human PDL tissues (a,b) and PDLSCs (c,d) from healthy controls (HC) and patients with diabetic periodontitis (DM+PD). Quantitative analysis was performed using ImageJ software (*n* = 3 per group). (e) Relative mRNA expression levels of *QRICH1* and ER stress‐related genes in hPDLSCs (*n* = 3 per group). (f) Transmission electron microscopy (TEM) images of ER morphology in hPDLSCs from HC, periodontitis without diabetes (PD, using independent patient samples), and DM+PD groups. Scale bars, 500 nm. (g) Relative mRNA expression levels of *Qrich1* in mouse liver, heart, and adipose tissues (*n* = 3 per group). (h,i) Relative mRNA expression levels of *Qrich1* and UPR‐related genes (*Grp78, Perk, Ire1α, Chop*) in mouse gingival tissues (*n* = 3 per group). (j) Immunofluorescence staining of QRICH1, GRP78, and Caspase‐3 in mouse periodontal tissues. Scale bars, 50 µm. (k‐m) Histological staining (H&E, Masson's trichrome) of periodontal tissues (k,l) and micro‐CT reconstruction of maxilla (m) in mice with periodontitis with or without diabetes (*n* = 5 mice per group). Scale bars, 200 µm, 50 µm, 1 mm. (n) Quantitative analysis of bone morphometric parameters, including bone volume fraction (BV/TV), trabecular thickness (Tb.Th), trabecular number (Tb.N), and trabecular separation (Tb.Sp) (*n* = 5 mice per group). HC: healthy control; PD: periodontitis; DM+PD: diabetic periodontitis; ab: alveolar bone; PDL: periodontal ligament; R: root. WT: wild‐type mice, db/db: diabetic mice (BKS background). Bar graphs: Values are presented as mean ± SEM. ns, not significant. ^*^ *p* < 0.05, ^**^ *p* < 0.01, ^***^ *p* < 0.001, ^****^ *p* < 0.0001.

To further evaluate the role of QRICH1, we established a mouse model of diabetic periodontitis (db/db+PD) and observed that QRICH1 exhibited higher expression in the liver and heart, with a similar trend in adipose tissue (Figure 1g). qPCR analysis showed substantial elevation of *Qrich1* in gingival tissues, accompanied by upregulation of UPR‐related genes (*Grp78, Perk, Ire1α, Chop*), confirming robust activation of ER stress in the db/db+PD group (Figure 1h,i). Immunofluorescence staining of periodontal sections also revealed significant upregulation of QRICH1, Caspase‐3, and GRP78 in the db/db+PD group (Figure 1j). Besides, H&E and Masson's trichrome staining showed the most extensive periodontal tissue disruption and alveolar bone loss in the db/db+PD group (Figure 1k,l). Consistently, micro‐CT analysis suggested marked alveolar bone resorption in diabetic periodontitis mice, evidenced by reduced bone volume/tissue volume ratio (BV/TV), trabecular number (Tb.N), and trabecular thickness (Tb.Th), along with increased trabecular spacing (Tb.Sp) (Figure 1m,n). These findings indicate that QRICH1 is significantly upregulated in diabetic periodontitis and modulates the activation of ER stress and apoptotic signaling.

### 2.2 High‐Glucose and Inflammatory Conditions Disrupt ER Homeostasis, Upregulate QRICH1 Expression, and Impair hPDLSCs Function

To mimic the diabetic periodontal microenvironment in vitro, we treated hPDLSCs with lipopolysaccharide (LPS) and high‐glucose (HG). Flow cytometry analysis using surface marker‐specific antibodies was performed to determine the stemness of hPDLSCs (Figure S2a). Cells were cultured with varying doses of glucose to determine the optimal concentration for HG treatment by observing ER stress‐related genes expression (Figure S2b). mRNA transcriptome sequencing (RNA‐seq) found 170 upregulated and 59 downregulated genes in LPS+HG‐treated cells versus controls (Figure S2c). Gene Ontology (GO) analysis of the differentially expressed genes (DEGs) highlighted significant participation of inflammatory response, oxidative stress, and apoptosis, as well as protein oligomerization and calcium homeostasis disruption, collectively indicating ER stress activation (Figure 2a). Gene set enrichment analysis (GSEA) also displayed abnormalities in protein localization and targeting to ER function following LPS and HG treatment (Figure 2b). TEM detection demonstrated that LPS and/or HG stimulation induced marked swelling and expansion of the ER morphology (Figure 2c). Meanwhile, confocal microscopy observed a significant increase in ER‐tracker red fluorescence following both LPS and HG treatment (Figure 2d). Furthermore, flow cytometry analysis revealed significant apoptosis in the LPS+HG group, as well as a significant increase in reactive oxygen species (ROS) levels (Figure 2e,f).

**FIGURE 2:**
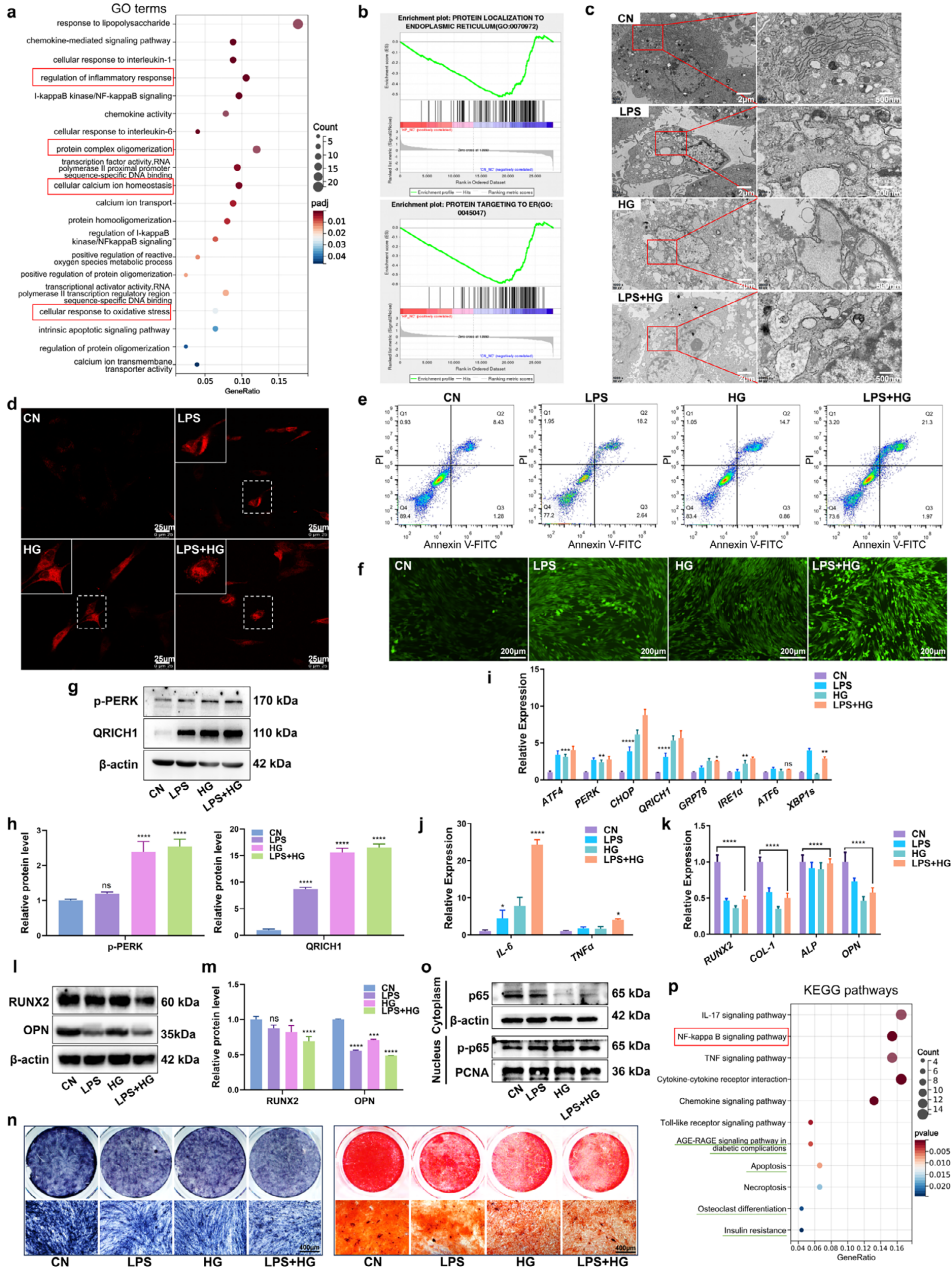
High‐glucose and inflammatory conditions disrupt ER homeostasis, upregulate QRICH1 expression, and impair hPDLSCs function. (a) GO enrichment analysis of DEGs in LPS+HG‐treated hPDLSCs compared to the control group revealed enhanced signaling associated with inflammation, apoptosis, and proteostasis. (b) GSEA analysis revealed abnormalities in protein localization and targeting to ER function in LPS+HG group cells. (c) TEM images of ER in different treatment groups. The red boxes indicate regions of interest showing ER morphology. Scale bars, 2 µm, 500 nm. (d) ER‐Tracker Red fluorescence was used to label and localize the ER in hPDLSCs treated with LPS and/or HG. Scale bars, 25 µm. (e) Apoptosis flow cytometry analysis revealed the apoptotic status of hPDLSCs across different groups. (f) Detection of intracellular ROS levels in hPDLSCs. Scale bars, 200 µm. (g,h) Western blot analysis of p‐PERK and QRICH1 protein expression in hPDLSCs following LPS and/or HG treatment and quantification using ImageJ software (*n* = 3 per group). (i,j) Relative mRNA expression levels of ER stress‐related genes (i) and representative inflammatory genes *IL‐6* and *TNF ‐ α* (j) in hPDLSCs (*n* = 3 per group). (k‐m) qPCR and western blot analyses of osteogenesis‐related genes and proteins expression (*n* = 3 per group). (n) ALP and Alizarin Red S staining showing the impact of LPS or LPS+HG stimulation on the osteogenic differentiation of hPDLSCs (*n* = 3 per group). Scale bars, 400 µm. (o) Isolation of nuclear proteins and western blot analysis of p‐p65 expression following LPS and/or HG treatment in hPDLSCs (*n* = 3 per group). (p) KEGG analysis of signaling pathway activation in hPDLSCs stimulated with LPS and HG. CN: controls; LPS: lipopolysaccharide; HG: high‐glucose. Bar graphs: Values are presented as mean ± SEM. ns, not significant. ^*^ *p* < 0.05, ^**^ *p* < 0.01, ^***^ *p* < 0.001, ^****^ *p* < 0.0001.

Critically, western blot analysis confirmed that LPS and HG treatment significantly upregulated QRICH1 expression and increased phosphorylation of PERK, a key ER stress sensor (Figure 2g,h). In addition to a significant increase in *QRICH1*, qPCR showed upregulation of multiple UPR‐related genes and inflammatory mediators (*IL‐6*, *TNF ‐ α*) (Figure 2i,j). However, the osteogenesis‐related genes and proteins were suppressed (Figure 2k–m), with attenuated cellular alkaline phosphatase (ALP) and diminished mineralized nodule production in Alizarin Red S (ARS) staining (Figure 2n). Similarly, these results were confirmed in the ER stress activation of hPDLSCs and 293T cells induced by tunicamycin (TM) (Figure S2d–m). Additionally, Kyoto Encyclopedia of Genes and Genomes (KEGG) analysis showed NF‐κB signaling was strongly activated in LPS+HG group, while pathways related to diabetes—including AGE‐RAGE signaling, apoptosis, osteoclast differentiation, and insulin resistance—also showed moderate upregulation (Figure 2p). Through nuclear‐cytoplasmic protein separation assays, we observed a significant accumulation of phosphorylated p65 in the nucleus of hPDLSCs following LPS and HG treatment, consistent with RNA‐seq results (Figure 2o).

### 2.3 QRICH1 Acts as a Key Effector Downstream of the UPR to Drive hPDLSCs Dysfunction

To investigate whether the expression and functional effects of QRICH1 depend on the UPR signaling, the ER stress inhibitor 4‐phenylbutyric acid (4‐PBA) was employed. Following exposure to LPS and HG, 4‐PBA treatment effectively suppressed the upregulation of QRICH1 and critical genes in the UPR pathway, particularly the PERK signaling axis (Figure 3a). Immunofluorescence analysis determined the subcellular localization of QRICH1, which is primarily expressed in the nucleus, and further demonstrated that inhibiting ER stress significantly attenuated its elevated expression under high‐glucose inflammatory conditions (Figure 3b). Meanwhile, at the protein level, we demonstrated that 4‐PBA not only reduced UPR markers such as GRP78, p‐PERK, QRICH1, XBP1s, and ATF6, but also significantly decreased the apoptotic executor cleaved caspase‐3 (Figure 3c and Figure S3a,b). Furthermore, the NF‐κB signaling marker p‐p65 was markedly attenuated (Figure 3d and Figure S3c).

**FIGURE 3:**
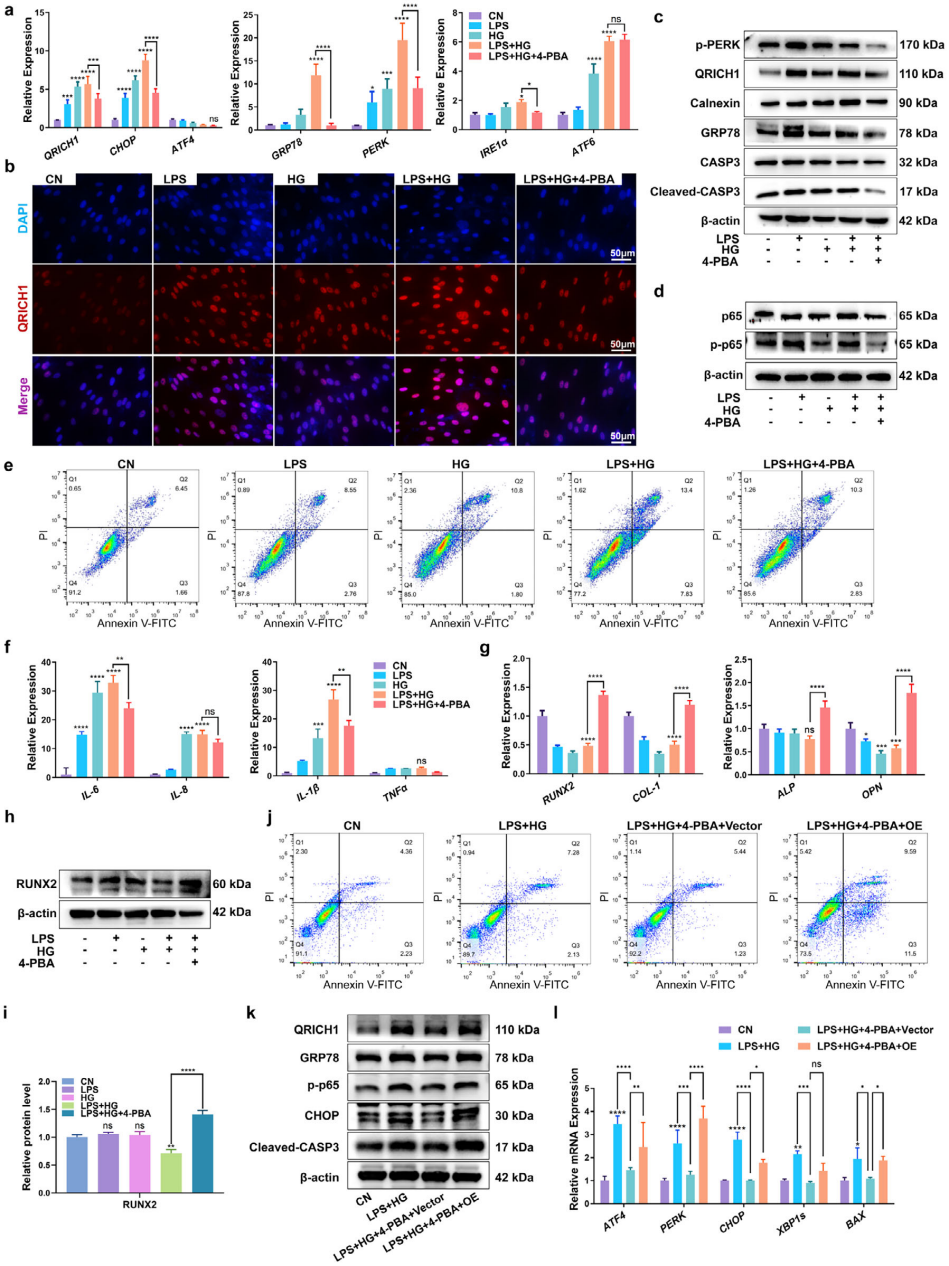
QRICH1 acts as a key effector downstream of the UPR to drive hPDLSCs dysfunction. (a) qPCR results indicated that 4‐PBA treatment of hPDLSCs significantly reduced the upregulation of key genes associated with the UPR pathway induced by LPS and/or HG (*n* = 3 per group). (b) Immunofluorescence staining revealed the expression and localization of QRICH1 in hPDLSCs from different groups. Scale bars, 50 µm. (c) Western blot analysis of QRICH1, ER stress‐related proteins, and the apoptotic marker cleaved caspase‐3 in hPDLSCs with 4‐PBA treatment. (d) Western blot analysis of p65 and p‐p65 expression. (e) Flow cytometry analysis revealed the apoptosis of hPDLSCs in different groups following 4‐PBA treatment. (f,g) qPCR analysis of the effect of 4‐PBA treatment on the expression of pro‐inflammatory cytokines and osteogenic marker genes in hPDLSCs (*n* = 3 per group).(h,i) Western blot analysis revealed expression of the osteogenic marker protein RUNX2 following 4‐PBA treatment and quantification using ImageJ software (*n* = 3 per group). (j) Flow cytometry analysis revealed that QRICH1 overexpression partially reverses the 4‐PBA‐mediated suppression of apoptosis in LPS+HG‐treated hPDLSCs. (k) Western blot analysis showed that overexpression of QRICH1 increased the expression of GRP78, CHOP, cleaved caspase‐3, and p‐p65 under LPS+HG+4‐PBA treatment. (l) qPCR analysis revealed that overexpression of QRICH1 increased the expression of ER stress‐related genes and *BAX*. CN: controls; LPS: lipopolysaccharide; HG: high‐glucose; 4‐PBA: 4‐phenylbutyrate; CASP3: caspase‐3. Bar graphs: Values are presented as mean ± SEM. ns, not significant. ^*^ *p* < 0.05, ^**^ *p* < 0.01, ^***^ *p* < 0.001, ^****^ *p* < 0.0001.

Functionally, alleviating ER stress with 4‐PBA markedly rescued hPDLSCs from LPS+HG‐induced injury. Flow cytometric analysis showed a substantial decrease in apoptosis rates (Figure 3e), and intracellular ROS production was also significantly reduced (Figure S3d). Furthermore, UPR inhibition resulted in a pronounced decline in *IL‐6* and *IL‐1β* (Figure 3f). Nevertheless, the impaired osteogenic potential following LPS+HG treatment was largely restored by 4‐PBA treatment, as evidenced by upregulated osteogenic gene and protein expression (Figure 3g–i), enhanced ALP staining, and increased matrix mineralization in ARS (Figure S3e,f).

To evaluate the functional necessity of QRICH1, we constructed a QRICH1 overexpression plasmid and validated its efficiency in hPDLSCs. Western blot and qPCR confirmed significant upregulation of QRICH1 protein and mRNA in the QRICH1 OE group compared to control or vector control (Figure S3g,h).

Then, to assess the impact of ER stress inhibition on QRICH1 function, we overexpressed QRICH1 in hPDLSCs and then treated the cells with 4‐PBA under LPS+HG conditions. Flow cytometry showed that QRICH1 overexpression partially reversed the 4‐PBA‐mediated suppression of apoptosis (Figure 3j). Consistently, western blot analysis revealed that QRICH1 overexpression markedly increased the protein levels of GRP78, CHOP, cleaved caspase‐3, and p‐p65, which had been suppressed by 4‐PBA (Figure 3k). qPCR further confirmed the elevated mRNA levels of *ATF4*, *PERK*, *CHOP*, and *BAX* under the same conditions (Figure 3l), indicating that QRICH1 counteracts the protective effects of ER stress inhibition on cellular stress and apoptotic signaling. Additionally, QRICH1 overexpression elevated the mRNA levels of *IL‑6* and *TNF‐α* under 4‑PBA treatment, further supporting its role in amplifying inflammation (Figure S3i). Furthermore, QRICH1 overexpression reduced ALP activity and matrix mineralization, counteracting the osteogenic rescue effect of 4‐PBA (Figure S3j,k).

These findings collectively demonstrate that QRICH1 is a critical downstream effector mediating unresolved ER stress‐induced hPDLSCs dysfunction.

### 2.4 QRICH1 Depletion Alleviates ER Stress and Restores hPDLSCs Function by Attenuating Global Protein Synthesis and NF‐κB Signaling

To demonstrate the critical role of QRICH1 in the UPR signaling pathway, we employed siRNA to knock down its expression following LPS+HG stimulation. First, we assessed the efficiency of si‐*QRICH1* knockdown (Figure 4a,b). RNA‐seq of si‐*QRICH1* cells displayed 215 upregulated and 89 downregulated genes compared to controls. Notably, the upregulated genes included several with potent protective functions, such as the pro‐osteogenic factor BMP2, the anti‐apoptotic gene *BTC*, and regulators of ER homeostasis *TRIM21* and *UBQLNL* (Figure 4c). Subsequently, GO analysis of downregulated genes showed significant enrichment in processes including amino acid synthesis and metabolism, the responses to the UPR, and calcium ion signaling (Figure 4d). KEGG analysis further confirmed that downregulated genes were closely associated with “amino acid biosynthesis” and “carbon metabolism” pathways (Figure 4e). These findings collectively indicate that *QRICH1* knockdown may alleviate ER stress by reducing the biosynthetic burden.

**FIGURE 4:**
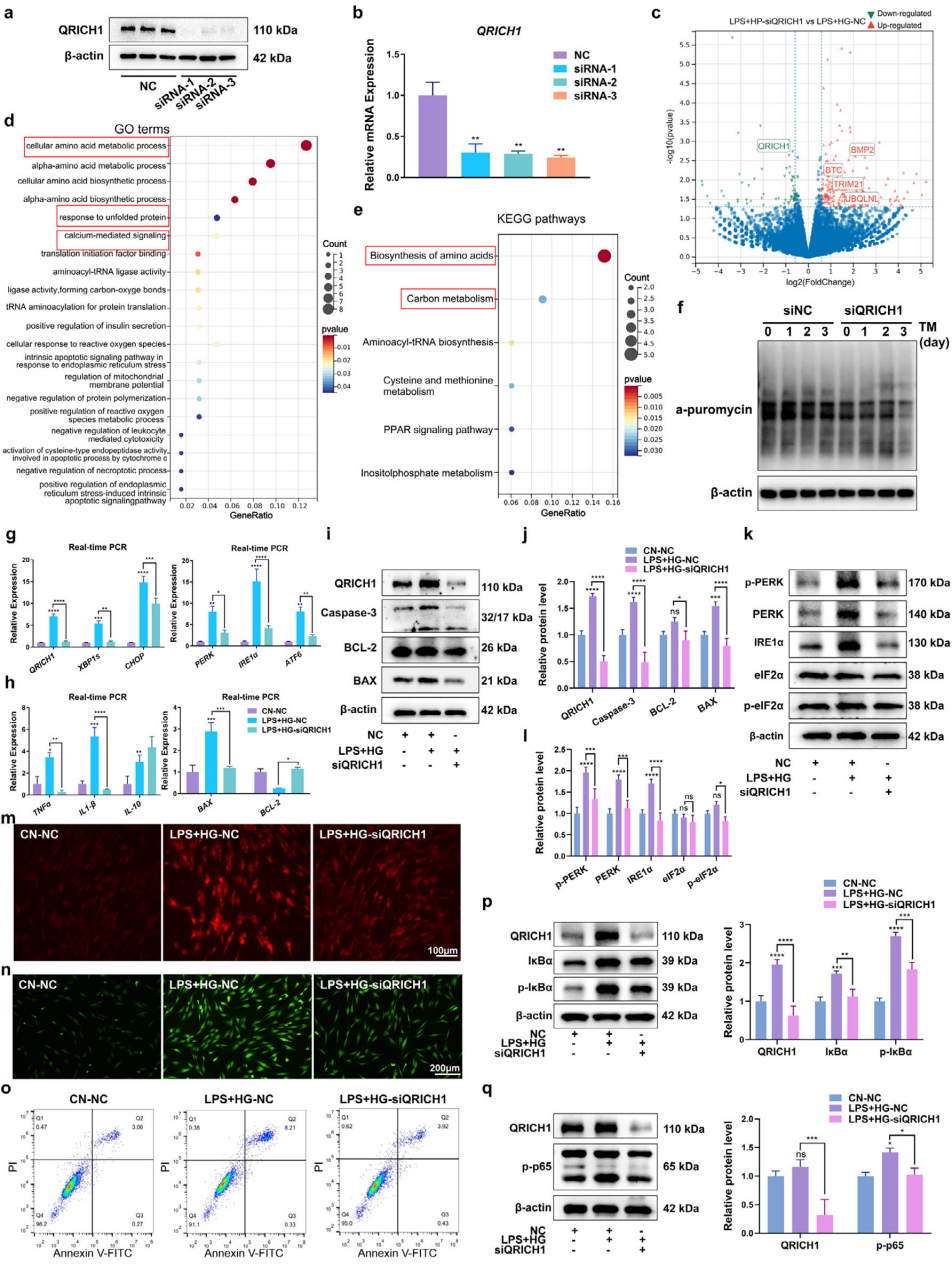
QRICH1 depletion alleviates ER stress and restores hPDLSCs function by attenuating global protein synthesis and NF‐κB signaling. (a,b) qPCR and western blot were used to assess the efficiency of si‐*QRICH1* knockdown (*n* = 3 per group). All three siRNAs significantly reduced QRICH1 expression, and siRNA‐1 was selected for further experiments. (c) Volcano plot of differentially expressed genes (DEGs) between hPDLSCs in LPS+HG‐siQRICH1 and LPS+HG‐NC groups, highlighting markers associated with osteogenesis, ER homeostasis, and anti‐apoptosis. (d) GO enrichment analysis of DEGs in LPS+HG‐siQRICH1 treated hPDLSCs compared to the LPS+HG‐NC group revealed decreased amino acid synthesis and metabolism, responses to the UPR, and calcium ion signaling. (e) KEGG analysis revealed that the “amino acid biosynthesis” and “carbon metabolism” signaling pathways were significantly downregulated in the LPS+HG‐siQRICH1 group. (f) The effect of *QRICH1* knockdown on global protein synthesis in hPDLSCs was assessed by puromycin incorporation assay (*n* = 3 per group). (g,h) Knockdown of *QRICH1* reduced the relative mRNA expression levels of ER stress‐related genes (g), representative inflammatory factors, and apoptosis markers (h) in LPS+HG‐treated hPDLSCs (*n* = 3 per group). (i–l) Western blot analysis and quantification revealed that *QRICH1* knockdown significantly reduced the expression of apoptosis markers (i,j) and ER stress‐related proteins (k,l) in LPS+HG‐stimulated hPDLSCs (*n* = 3 per group). (m) ER‐Tracker Red assay revealed a significant reduction in ER fluorescence intensity in the LPS+HG‐siQRICH1 group. Scale bars, 100 µm. (n) Following *QRICH1* knockdown, LPS+HG‐stimulated hPDLSCs exhibited a marked reduction in intracellular ROS production. Scale bars, 200 µm. (o) Flow cytometry analysis revealed decreased apoptosis in hPDLSCs under high‐glucose inflammatory conditions following *QRICH1* knockdown. (p,q) Western blot analysis and quantification with ImageJ demonstrated that *QRICH1* knockdown reduced the expression of NF‐κB signaling pathway‐associated proteins in a high‐glucose inflammatory environment (*n* = 3 per group). CN: controls; LPS: lipopolysaccharide; HG: high‐glucose; NC: negative control. Bar graphs: Values are presented as mean ± SEM. ns, not significant. ^*^ *p* < 0.05, ^**^ *p* < 0.01, ^***^ *p* < 0.001, ^****^ *p* < 0.0001.

We then performed puromycin incorporation assays to assess cellular protein synthesis rates. The results demonstrated that QRICH1 regulated translation, with its depletion leading to a significant reduction in global protein synthesis (Figure 4f). Additionally, *QRICH1* knockdown in a high‐glucose inflammatory environment resulted in the downregulation of UPR‐associated genes, suppression of the inflammatory factors *TNF ‐ α* and *IL‐1β*, reduced expression of the apoptotic factor *BAX*, and increased the anti‐apoptotic gene *BCL‐2* (Figure 4g,h). Western blot analysis also demonstrated decreased levels of ER stress and apoptosis following *QRICH1* knockdown (Figure 4i–l). Consistent with the alleviation of proteostatic stress, we observed attenuated ER‐Tracker signal intensity and diminished intracellular ROS accumulation (Figure 4m,n). Flow cytometry assays also demonstrated decreased cell apoptosis (Figure 4o). Furthermore, we detected a marked decrease in phosphorylated IκBα and p65 proteins upon *QRICH1* knockdown, indicating that excessive activation of NF‐κB signaling was substantially suppressed (Figure 4p,q). The effect of *QRICH1* knockdown on the UPR signaling pathway was further validated by TM stimulation of hPDLSCs (Figure S4a–c).

### 2.5 QRICH1 Interacts With NF‐κB, Forming a Positive Feedback Loop That Exacerbates the UPR

Co‐immunoprecipitation (Co‐IP) analysis in hPDLSCs and 293T cells revealed a previously unreported interaction between p65 and QRICH1, providing a mechanistic basis for QRICH1‐mediated NF‐κB activation and cellular dysfunction (Figure 5a and Figure S5a). To precisely characterize this interaction, we constructed a molecular docking model, which predicted the optimal binding interface between QRICH1 and NF‐κB, and highlighted key residues hydrophobic interactions (Figure 5b). Molecular dynamics simulations demonstrated that the NF‐κB protein exhibited a higher root‐mean‐square deviation (RMSD), indicating greater conformational dynamics and structural flexibility than QRICH1 during the simulation (Figure S5b). Energetic analysis identified ASP‐204 of QRICH1 and ASP‐707 of NF‐κB as the most critical residues driving the interaction (Figure S5c). Crucially, the calculated protein‐protein binding free energy (ΔG = ‐17.01 kcal/mol) suggested that the QRICH1‐NF‐κB complex formation is a thermodynamically favorable, spontaneous process with high affinity (Figure 5c).

**FIGURE 5:**
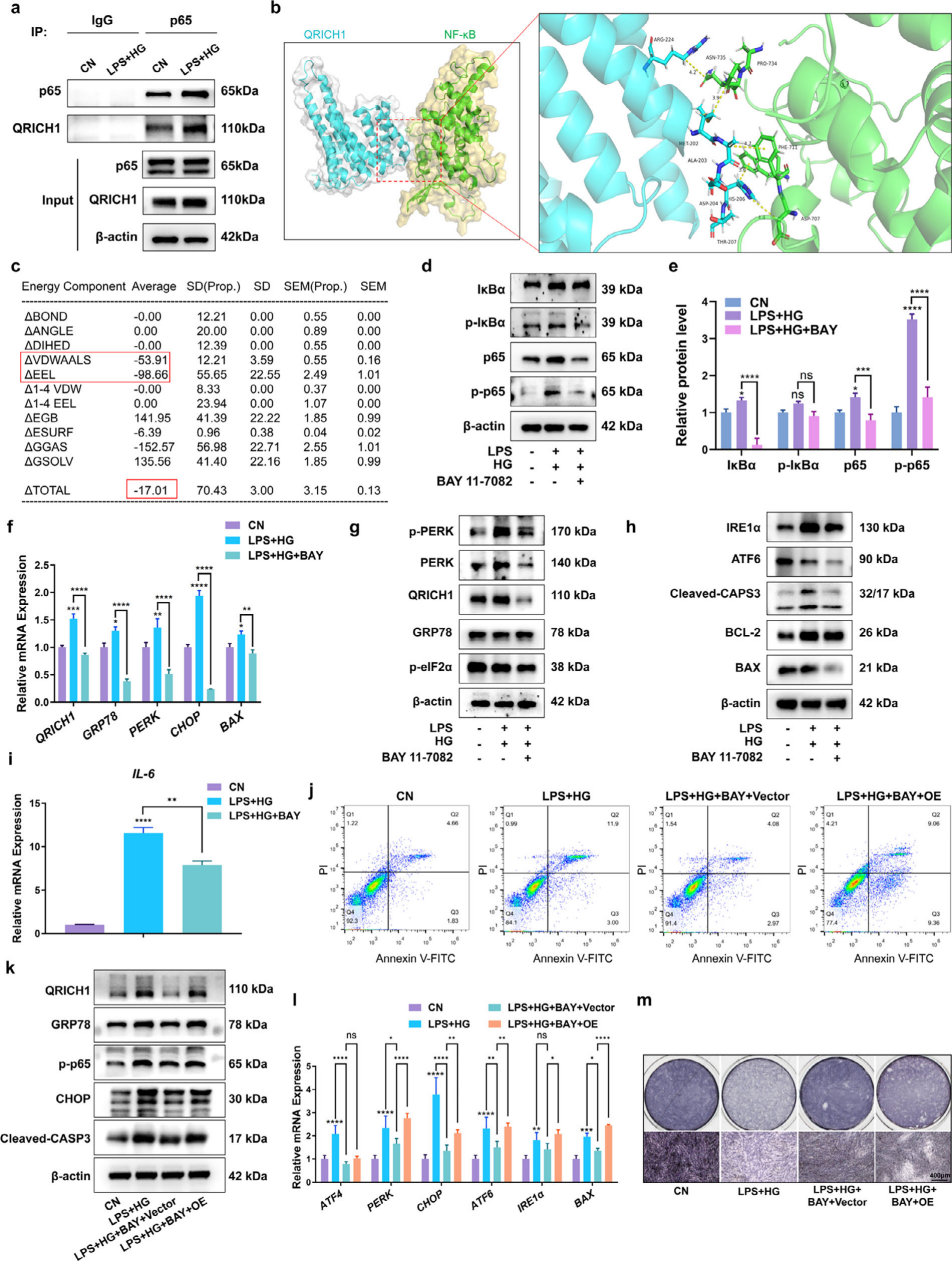
QRICH1 interacts with NF‐κB to form a positive feedback loop that exacerbates the UPR. (a) Co‐immunoprecipitation analysis revealed enhanced interaction between p65 and QRICH1 in LPS+HG‐treated hPDLSCs (*n* = 3 per group). (b) A molecular docking model predicted the optimal binding interface between QRICH1 and NF‐κB. The region of interest was outlined in the red dashed box, and the magnified image displayed the hydrophobic interactions of key residues. (c) Calculated protein‐protein binding free energy (ΔG = ‐17.01 kcal/mol) from molecular dynamics simulations of QRICH1 and NF‐κB. (d,e) Western blot analysis and quantification with ImageJ showed that BAY 11–7082 significantly suppressed the phosphorylation of IκBα and p65 in hPDLSCs (*n* = 3 per group). (f) qPCR analysis demonstrated that inhibition of NF‐κB signaling significantly lowered the expression of QRICH1, ER stress‐associated genes, and apoptosis marker *BAX* (*n* = 3 per group). (g,h) Western blot analysis showed that inhibition of the NF‐κB signaling pathway markedly reduced the expression of QRICH1, UPR‐associated proteins, and apoptotic proteins in hPDLSCs. (i) qPCR analysis of *IL‐6* with BAY 11–7082 treatment (*n* = 3 per group). (j) Flow cytometry analysis revealed that QRICH1 overexpression partially reverses the anti‐apoptotic effect of BAY 11–7082, with an increase in apoptotic cells compared to the LPS+HG+BAY+Vector group. (k) Western blot analysis showed that QRICH1 overexpression restores p‐p65 levels as well as the expression of GRP78, CHOP, and cleaved caspase‐3 under BAY 11–7082 treatment. (l) qPCR analysis demonstrated that QRICH1 overexpression increases mRNA levels of ER stress‐related genes (*PERK*, *ATF6*, *CHOP*), and the apoptotic gene *BAX* under BAY 11–7082 treatment (*n* = 3 per group). (m) ALP staining indicated that QRICH1 overexpression counteracts the osteogenic rescue effect of NF‐κB inhibition. Scale bars, 400 µm. CN: controls; LPS: lipopolysaccharide; HG: high‐glucose; BAY: BAY 11–7082; OE: overexpression; ALP: alkaline phosphatase. Bar graphs: Values are presented as mean ± SEM. ns, not significant. ^*^ *p* < 0.05, ^**^ *p* < 0.01, ^***^ *p* < 0.001, ^****^ *p* < 0.0001.

We next investigated the functional consequences of this interaction. After optimizing the concentration using a CCK‐8 viability assay, hPDLSCs were treated with an NF‐κB pathway inhibitor (BAY 11–7082) (Figure S5d). Western blot analysis suggested that the inhibitor significantly decreased phosphorylation of IκBα and p65 under a high‐glucose inflammatory microenvironment (Figure 5d,e). Subsequently, it markedly downregulated QRICH1 expression at both mRNA and protein levels (Figure 5f,g). This inhibition cascade simultaneously resulted in a marked reduction in UPR‐related molecules, pro‐inflammatory cytokines, and pro‐apoptotic signals (Figure 5f–i and Figure S5e,f).

Therefore, to further verify whether QRICH1 functionally enhances NF‐κB signaling through a positive feedback mechanism, we overexpressed QRICH1 under LPS+HG conditions in the presence of BAY 11–7082. Flow cytometry analysis indicated that QRICH1 overexpression partially reversed the anti‐apoptotic effect of BAY 11–7082, with an increase in the number of apoptotic cells compared to the LPS+HG+BAY+Vector group (Figure 5j). Consistently, western blot analysis showed that QRICH1 overexpression restored p‐p65 levels, as well as the expression of GRP78, CHOP, and cleaved caspase‐3 (Figure 5k). qPCR further demonstrated that, under BAY treatment conditions, QRICH1 overexpression increased mRNA levels of ER stress‐related genes *PERK*, *ATF6*, *CHOP*, the apoptotic gene *BAX*, and the inflammatory genes *IL‐6* (Figure 5l and Figure S5g). Furthermore, ALP and Alizarin Red S staining indicated that QRICH1 overexpression counteracted the osteogenic rescue effect produced by NF‐κB inhibition (Figure 5m and Figure S5h).

In summary, these findings reveal a hitherto undocumented interaction between QRICH1 and NF‐κB that exacerbates hPDLSC dysfunction in diabetic periodontitis via the UPR pathway. Moreover, our rescue experiments demonstrate that QRICH1 actively amplifies NF‐κB signaling, creating a self‐reinforcing pathogenic cycle in diabetic periodontitis.

### 2.6 In Vivo Knockdown of *Qrich1* Ameliorates Diabetic Periodontitis and Suppresses the ER Stress‐NF‐κB Axis

To further elucidate the pathogenic role of QRICH1, we employed adeno‐associated virus (AAV) for gene knockdown in a diabetic periodontitis mouse model. Knockdown efficiency of AAV‐mediated *Qrich1* was assessed by analyzing the mRNA and protein levels in gingival tissues (Figure 6a,b). Micro‐CT showed that AAV‐mediated *Qrich1* knockdown significantly rescued the severe alveolar bone loss observed in the WT‐PD and db/db‐PD groups (Figure 6c), as evidenced by increased BV/TV, Tb.Th, and Tb.N, yet decreased Tb.Sp (Figure 6d,e). H&E staining revealed that *Qrich1* depletion not only restored alveolar bone height but also promoted a more organized arrangement of periodontal ligament collagen fibers and markedly reduced inflammatory cell infiltration within the junctional epithelium (Figure 6f).

**FIGURE 6:**
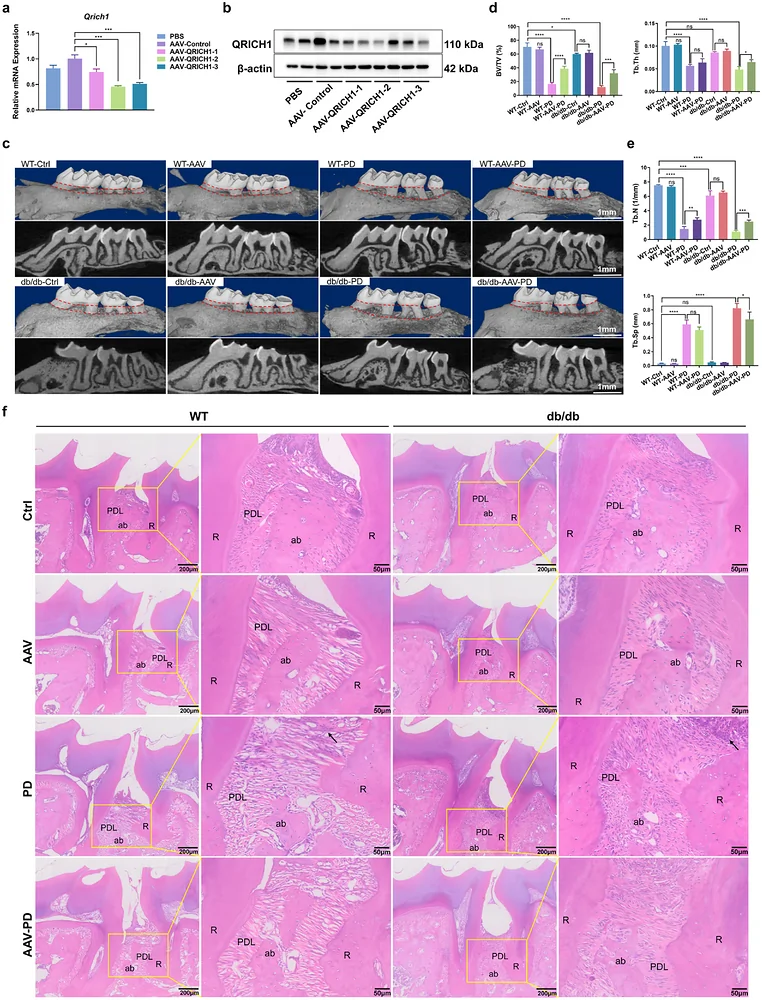
Knockdown of *Qrich1* improves bone defects in diabetic periodontitis mice. (a, b) qPCR and western blot were used to assess the efficiency of AAV‐mediated *Qrich1* knockdown (*n* = 3 per group). (c) Micro‐CT reconstruction of maxilla in WT and db/db mice with or without AAV‐mediated *Qrich1* treatment (*n* = 5 mice per group). Scale bars, 1 mm. (d,e) Quantitative analysis of bone morphometric parameters, including BV/TV, Tb.Th, Tb.N, and Tb.Sp (n = 5 mice per group). (f) Hematoxylin and eosin staining of periodontal tissues from different groups of mice. Scale bars, 200 µm, 50 µm. AAV: adeno‐associated virus; PD: periodontitis; ab: alveolar bone; PDL: periodontal ligament; R: root. WT, db/db mice (BKS background). Bar graphs: Values are presented as mean ± SEM. ns, not significant. ^*^ *p* < 0.05, ^**^ *p* < 0.01, ^***^ *p* < 0.001, ^****^ *p* < 0.0001.

Masson's trichrome staining further confirmed improved collagen fiber density and tissue architecture (Figure 7a). Furthermore, immunohistochemical analysis detected a substantial increase in caspase‐3 positive cells in the db/db and periodontitis groups, which was effectively reduced following AAV‐mediated *Qrich1* pretreatment (Figure 7b). Immunofluorescence staining demonstrated that *Qrich1* knockdown also suppressed the robust upregulation of p65 induced by diabetes and periodontitis (Figure 7c). Finally, multiplex immunofluorescence analysis provided a comprehensive molecular picture, indicating that in vivo ablation of *Qrich1* concurrently reduced the expression of p65 and caspase‐3, as well as key UPR‐associated proteins including GRP78, PERK, and CHOP (Figures 8 and 9). Consistently, western blot analysis showed that knocking down *Qrich1* in diabetic periodontitis mice reduced the expression of PERK, GRP78, and the apoptotic marker cleaved caspase‐3, while also decreasing p65 phosphorylation (Figure S6).

**FIGURE 7:**
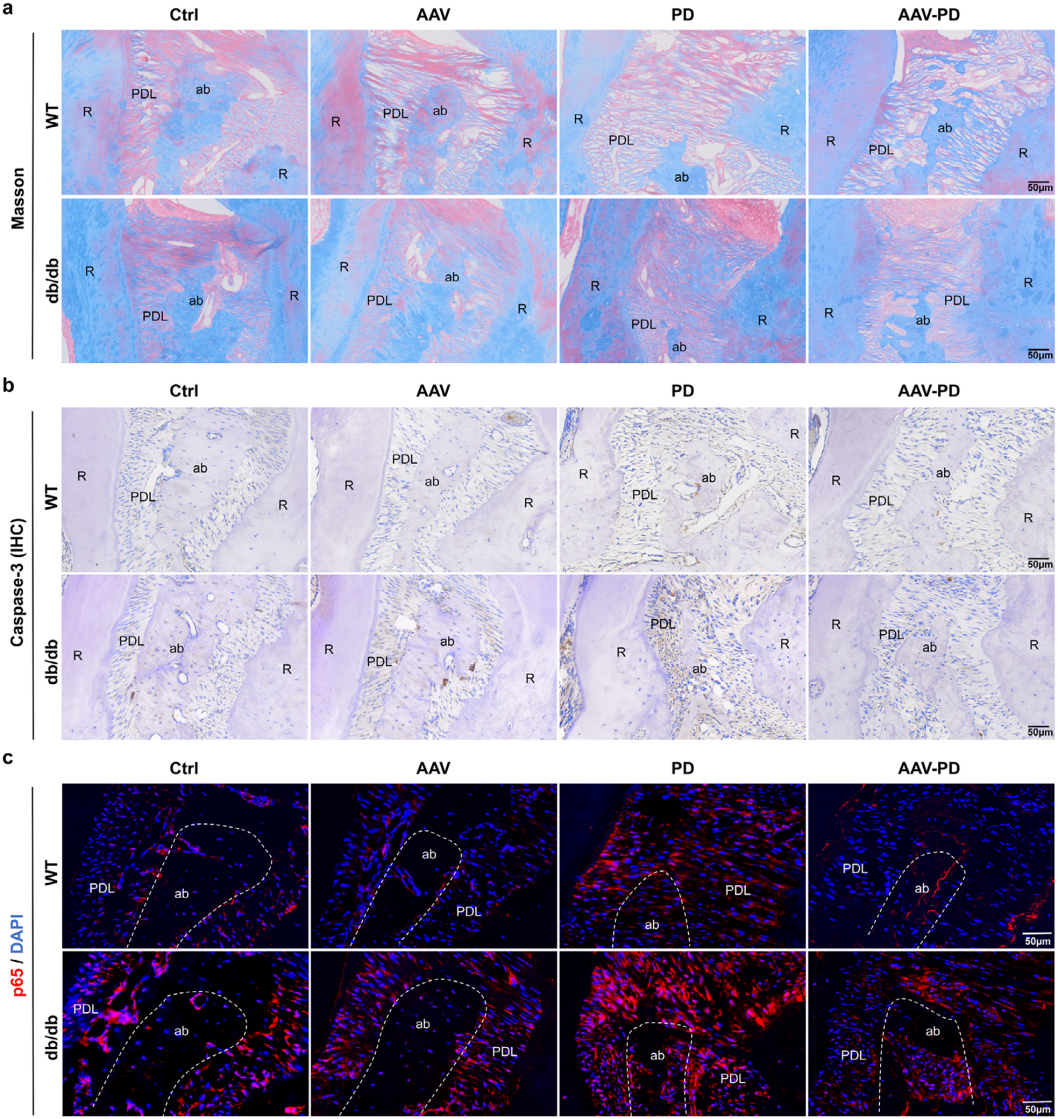
Histological analysis of periodontal tissues in AAV‐mediated *Qrich1* knockdown mice. (a) Masson's trichrome staining of periodontal tissues from different groups of mice. Scale bars, 50 µm. (b) Immunohistochemical staining analysis of the effect of AAV‐mediated *Qrich1* knockdown on caspase‐3 expression. Scale bar, 50 µm. (c) Immunofluorescence staining analysis detected p65 expression in periodontal tissues of mice from different groups. Scale bar, 50 µm. AAV: adeno‐associated virus; PD: periodontitis; ab: alveolar bone; PDL: periodontal ligament; R: root. WT, db/db mice (BKS background).

**FIGURE 8:**
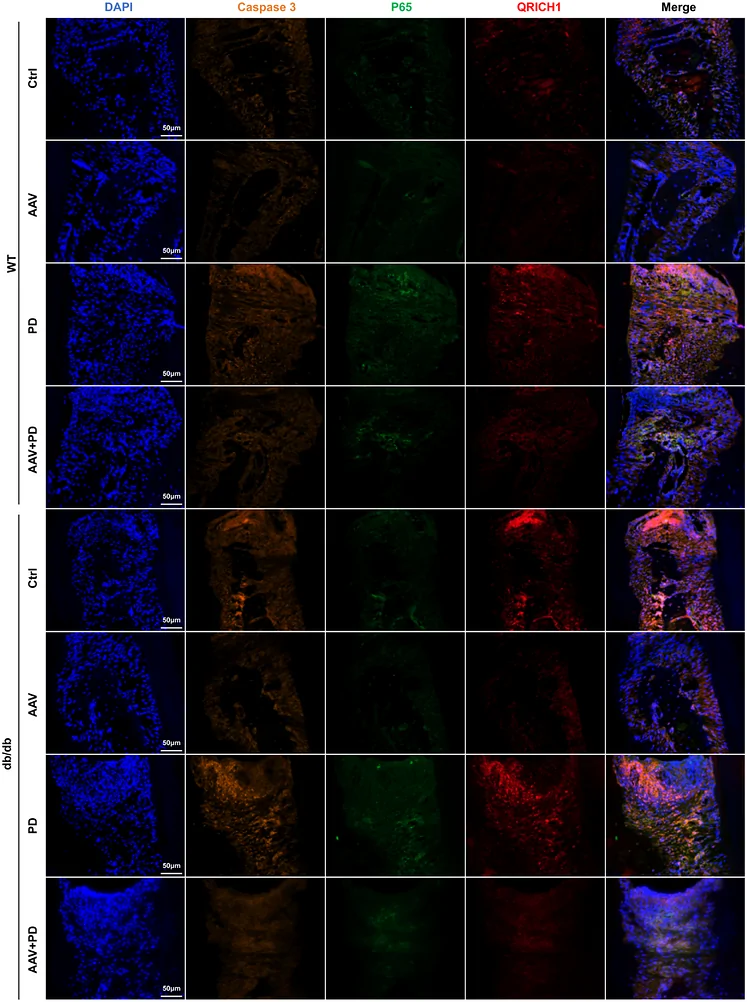
In vivo knockdown of *Qrich1* suppresses the NF‐κB signaling and apoptosis. Multiplex immunofluorescence analysis revealed that AAV‐mediated *Qrich1* knockdown reduced the expression of caspase‐3 and p65 in the periodontal tissues of diabetic periodontitis mice. Scale bars, 50 µm. AAV: adeno‐associated virus; PD: periodontitis. WT, db/db mice (BKS background).

**FIGURE 9:**
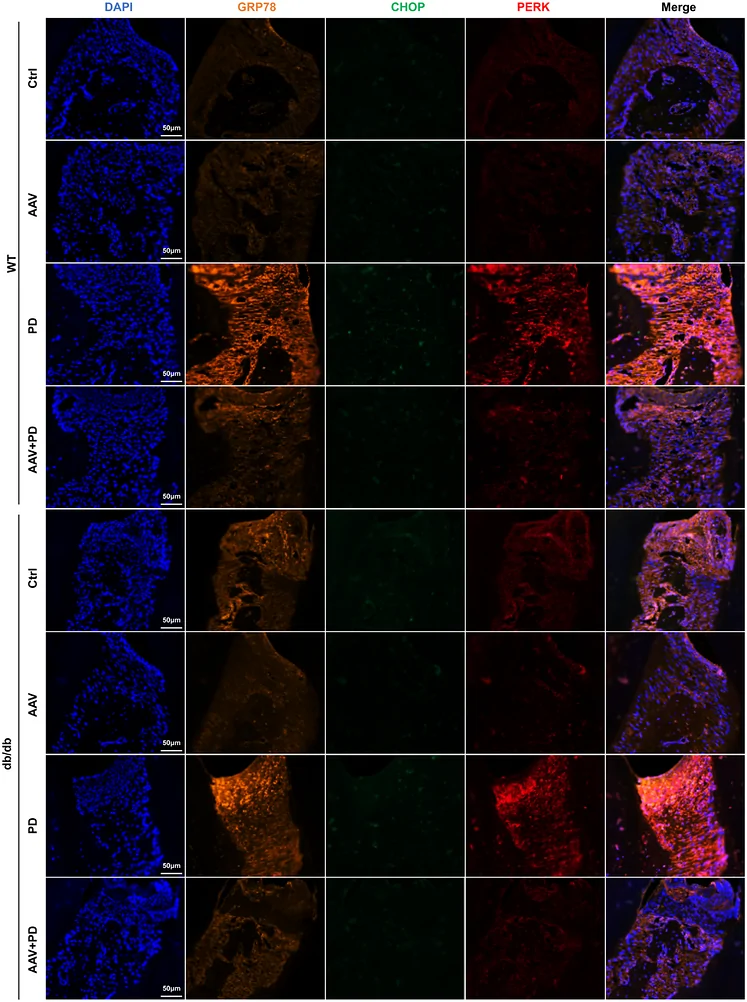
In vivo knockdown of *Qrich1* alleviates the ER stress. Multiplex immunofluorescence results indicated that AAV‐mediated *Qrich1* knockdown reduced ER stress‐related markers (GRP78, CHOP, PERK) expression in the periodontal tissues of diabetic periodontitis mice. Scale bars, 50 µm. AAV: adeno‐associated virus; PD: periodontitis. WT, db/db mice (BKS background).

## 3 Discussion

The ER is pivotal for protein biosynthesis, folding, and calcium homeostasis, with its functional integrity being essential for cellular health [46]. Various physiological and pathological stressors can trigger ER stress and UPR by promoting the aggregation of misfolded or unfolded proteins [15]. However, persistent and unresolved UPR may ultimately lead to cell death and functional impairment [47, 48]. The precise molecular mechanisms by which this adaptive program in diabetic periodontitis transforms into maladaptive drivers leading to periodontal tissue dysfunction have not been fully elucidated. In this study, we establish that diabetic periodontitis triggers the upregulation of QRICH1 via UPR activation, particularly through the PERK‐eIF2α axis. Moreover, QRICH1 induces functional impairment in hPDLSCs by enhancing global protein synthesis, forming a positive feedback loop via direct interaction with NF‐κB, and ultimately exacerbating apoptosis and periodontal tissue destruction. Genetic knockdown of *QRICH1* effectively disrupts this pathological cycle, thereby rescuing cellular function and mitigating disease pathology. These findings collectively establish QRICH1 as a novel effector of UPR‐driven periodontal injury in diabetic periodontitis, identifying it as a crucial mediator of disease progression and a potential therapeutic target, as illustrated in the schematic model in Figure 10.

**FIGURE 10:**
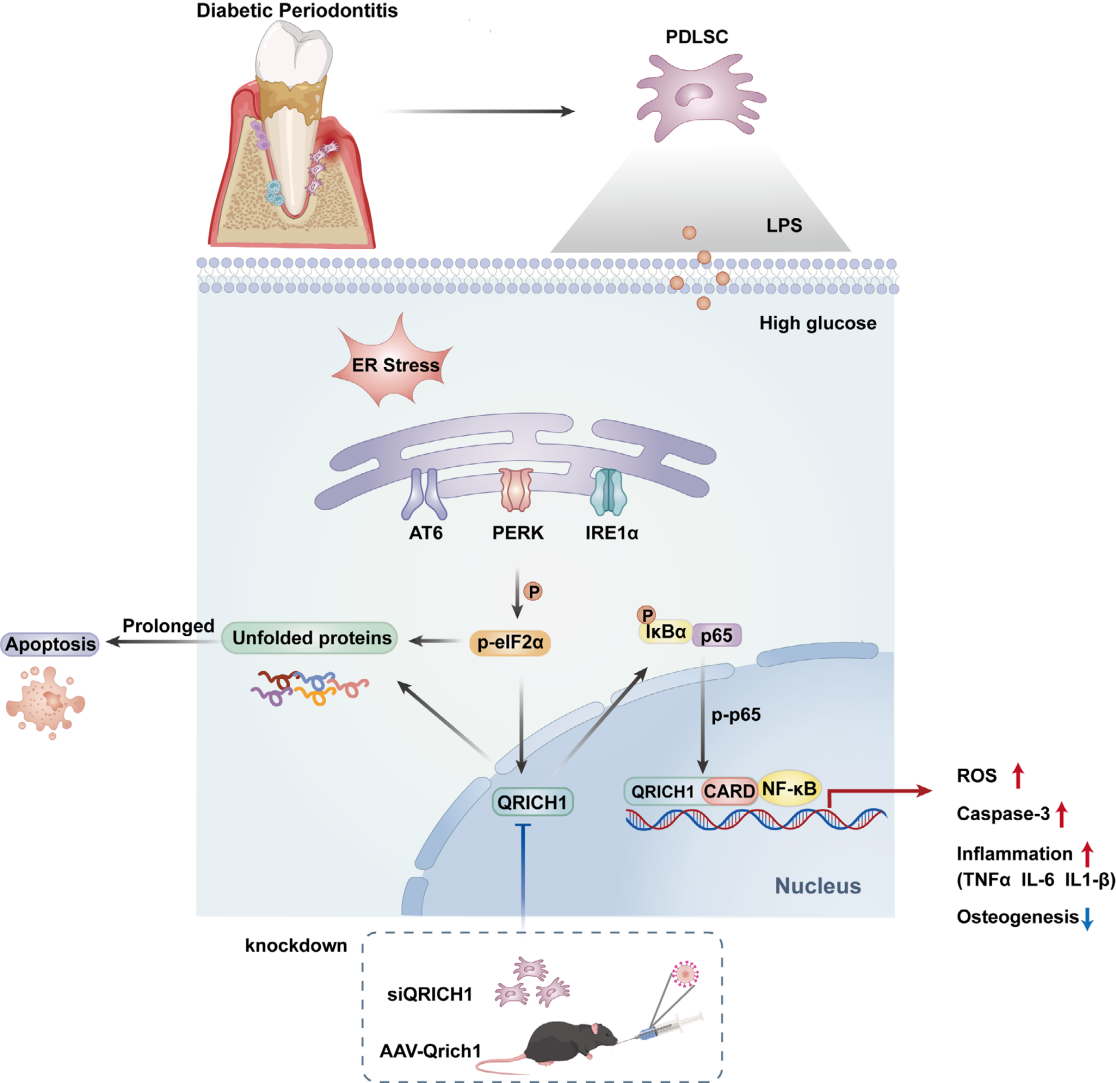
Schematic diagram of this study. The diabetic periodontitis microenvironment induces persistent and unresolved ER stress in hPDLSCs. QRICH1, a key downstream molecule of the UPR (PERK‐eIF2α axis), promotes global protein synthesis and forms a positive feedback loop with NF‐κB through direct interaction. This self‐amplifying “stress‐inflammation amplifier” ultimately leads to functional collapse of hPDLSCs and accelerated periodontal destruction.

The UPR exerts context‐dependent dual functions in stem cells: mild or transient activation promotes adaptation and survival by restoring ER homeostasis, whereas unresolved chronic activation triggers pro‐apoptotic pathways and impairs stem cell function [49]. Within this process, the fine‐tuning of the PERK axis plays a pivotal role in determining cell fate [50]. Upon activation, PERK phosphorylates eIF2α, initially aiding in achieving ER protein homeostasis by decreasing the flux of client proteins [51]. For instance, activating the PERK‐ATF4‐WARS1 axis during early cardiac differentiation reduces misfolded protein aggregation and alleviates protein toxicity stress in human pluripotent stem cells [52]. However, during prolonged ER stress, the activation of amino acid biosynthesis and secretory pathways promotes the recovery of translational activity and facilitates apoptosis [29, 53, 54]. Previous studies have shown that an overactivated UPR can be detrimental. For example, ER stress in the context of obesity activates the PERK pathway and attenuates adipocyte differentiation of adipose‐derived stem cells [55]. Zhang et al. found that the UPR in aged PDLSCs suppresses their stemness via the ATF6/p53 axis [18]. Besides, the long‐term inflammatory factors in periodontitis induced sustained ER stress and reduced the osteogenic potential of PDLSCs [20]. Overall, these findings emphasize the detrimental effects of UPR dysfunction on cellular function. Consistent with this, our study confirms that under high‐glucose inflammatory conditions, the UPR (especially PERK branch) in hPDLSCs is strongly activated, promoting cell apoptosis and inflammatory response by significantly upregulating QRICH1 expression, while inhibiting osteogenic activity. QRICH1 induces proteotoxicity and terminal UPR by increasing intracellular protein load. A recent study revealed that QRICH1 serves as a key downstream signaling molecule of the PERK axis, governing a transcriptional program linked to translational and secretory networks that is particularly upregulated in inflammatory pathologies [31]. Precise regulation of protein synthesis is crucial for stem cells to maintain normal function [56]. Our results indicate that knocking down *QRICH1* under high‐glucose inflammatory conditions reduces amino acid biosynthesis and protein synthesis. This attenuation of translational flux subsequently alleviates ER proteotoxicity, thereby restoring cellular homeostasis and osteogenic capacity in diabetic periodontitis. Although our study focused primarily on the PERK‐QRICH1 axis, our data also indicate that the IRE1α and ATF6 pathways are activated under diabetic inflammatory stress. A recent study showed that QRICH1 enhances ATF6 transcription in cardiomyocytes, exacerbating cardiac dysfunction and inflammation [34]. Currently, direct evidence linking QRICH1 to IRE1α is limited, but our results suggest that IRE1 signaling is at least partially regulated through the UPR network mediated by QRICH1. Future ChIP‐seq or CUT&Tag studies in relevant cell types could clarify whether QRICH1 directly binds to the promoters of IRE1α, ATF6, or other UPR‐related genes, thereby fully elucidating how QRICH1 coordinates signaling across multiple UPR pathways in diabetic periodontitis.

Beyond its role in transcriptional regulation, QRICH1 functions as a critical node in protein‐protein interaction networks, forming multi‐molecular complexes to modulate cellular behavior. For instance, QRICH1 binds to CARD11 to regulate NF‐κB signaling, thereby influencing the activation and proliferation of CD8+ T cells [32]. Additionally, it interacts with GRP78 to promote apoptosis in T‐ALL cells [33], and forms the Zincore complex with SEPHS1, a requirement for embryonic development [57]. By binding to distinct molecular partners, QRICH1 may function as a versatile node that integrates stress and inflammatory signals. Notably, our work demonstrated binding between QRICH1 and the p65 subunit through co‐IP assays, and the predicted molecular docking model and molecular dynamics simulations indicated a high‐affinity physical interaction between QRICH1 and NF‐κB. Furthermore, *QRICH1* knockdown significantly reduced p65 phosphorylation, downregulated *TNF ‐ α* and *IL‐1β* expression, and restored cellular function. These findings suggest that QRICH1 may function as a cofactor or stabilizer, directly amplifying the inflammatory effects of NF‐κB signaling. Our research has revealed that the QRICH1/NF‐κB linkage effectively creates a “stress‐inflammation amplifier” that drives inflammation and periodontal tissue destruction in diabetic periodontitis. However, whether other proteins are involved and the precise molecular details of this binding warrant further exploration. Notably, QRICH1 expression levels are elevated in the liver and heart, and a similar trend has been observed in adipose tissue, suggesting that the significance of QRICH1 extends beyond the local context of periodontal pathology. In fact, chronic ER stress and activation of the UPR are key features of diabetes development; they are not limited to periodontal tissues in diabetic states but also occur in the liver and adipose tissue [58, 59]. The upregulation of QRICH1 in multiple tissues may suggest that its regulatory mechanism in ER stress is a universal cell biological mechanism. While our focus is on the periodontal tissue, further interdisciplinary research is needed to elucidate the specific mechanisms at play in other tissues. Future systematic mapping of the QRICH1 interactome using approaches such as ChIP‑seq or co‑immunoprecipitation‑mass spectrometry will be crucial to fully elucidate its regulatory network and its context‑dependent roles in different diseases.

Furthermore, diabetic periodontitis is not merely the combined result of hyperglycemia and bacterial inflammation; rather, metabolic stress can reshape the host responsiveness to microbial periodontal biofilms [60]. Although we identified key inflammatory signals using in vitro models involving LPS and high‐glucose, these models do not fully capture the polymicrobial environment of periodontal biofilms or the dynamic interactions between resident immune cells and periodontal tissues. The QRICH1‐NF‐κB feedback loop identified in our study represents a self‐amplifying pathogenic cycle that drives inflammation and tissue destruction. A key advantage of this mechanism is the identification of QRICH1 as a central node that integrates ER stress and inflammatory signaling, suggesting that targeting QRICH1 could simultaneously alleviate proteotoxic stress and chronic inflammation. However, targeting QRICH1 does not directly eliminate mixed‐bacterial biofilms or completely reset immune cell‐derived inflammatory memory; thus, future studies involving bacteriologically defined or mixed‐bacterial models of diabetic periodontitis and immune cell‐resolved analyses are necessary. Additionally, while our in vivo model employed AAV‐mediated knockdown, future studies utilizing cell‐type‐specific approaches will help elucidate the role of QRICH1 in different periodontal cell populations and rule out potential off‐target effects that may limit therapeutic specificity.

Local delivery of AAV‐mediated gene therapy is a promising tool for treating periodontal diseases [61]. Studies have demonstrated that AAV vectors can achieve local gene knockdown, thereby enabling specific regulation of single or multiple genes in vivo, with extensive application in both experimental and clinical settings [62, 63, 64]. For instance, AAV‐mediated suppression of *cathepsin K* expression in mice alleviates bone destruction in periodontitis with rheumatoid arthritis [65]. Moreover, local injection of AAV‐*Rgs10* into bacterial‐induced inflammatory tissues protects periodontal structures from inflammatory injury and bone destruction [66]. It has been reported that AAV‐mediated *Qrich1* knockdown reduces ER stress‐associated protein expression and neuronal death, thereby alleviating secondary brain injury [35]. Similarly, in our work, AAV‐mediated *Qrich1* knockdown rescued alveolar bone loss and reduced inflammatory cell infiltration in diabetic periodontitis mice. Concurrently, UPR markers, NF‐κB activation, and apoptosis markers were downregulated. Based on our findings, we propose that AAV‐mediated *Qrich1* knockdown could serve as a novel and effective therapeutic approach for diabetic periodontitis, yet its long‐term safety requires further investigation.

## 4 Conclusion

In conclusion, our work establishes QRICH1 as a master regulatory node that critically links unresolved ER stress to sustained inflammatory signaling in diabetic periodontitis. We demonstrated that PERK‐eIF2α axis‐induced QRICH1 drives a maladaptive program by promoting global protein synthesis and engaging in a positive feedback loop with NF‐κB via direct interaction with p65. This self‐reinforcing “stress‐inflammation amplifier” ultimately leads to the functional collapse of hPDLSCs and accelerated periodontal breakdown. Our findings not only deepen the molecular understanding of diabetic periodontitis but also identify QRICH1 as a promising therapeutic target to disrupt the vicious cycle between proteotoxic stress and chronic inflammation.

## 5 Experimental Section

### 5.1 Human Periodontal Ligament Tissue Collection and Cell Culture

The PDL tissues were obtained from extracted teeth of patients aged 25–45 years. Healthy controls (HC) with no evidence of attachment loss or periodontitis were included. Patients with diabetic periodontitis (DM+PD) were required to meet the diagnostic criteria for Type 2 Diabetes Mellitus (T2DM) and exhibit clinical manifestations of periodontitis, including probing depths exceeding 6 mm, bleeding on probing, and radiographic evidence of alveolar bone loss [67, 68]. Exclusion criteria for all participants included a history of smoking, the presence of other systemic diseases, and pregnancy. Harvested PDL tissues (the middle third of the root) were placed in liquid nitrogen (−80°C storage) for subsequent protein analysis. To establish primary cell cultures from healthy individuals (HC) and periodontitis patients with or without diabetes (PD; DM+PD), freshly extracted teeth were thoroughly cleaned, and PDL tissues were harvested via mechanical scraping. The isolated tissues were subsequently subjected to enzymatic digestion using type I collagenase (3 mg/mL, Sigma‐Aldrich, USA) at 37°C with gentle agitation for half an hour. After centrifugation, tissues were incubated in α‐MEM medium (HyClone, USA) supplemented with 10% fetal bovine serum (FBS, Gemini, USA) and 1% penicillin‐streptomycin (HyClone, USA) in a 37°C incubator with 5% CO_2_. Cells were digested and passaged when confluency approached nearly 90%, and passages 1–3 were used for subsequent western blot, qPCR, and TEM assays.

### 5.2 Animals

BKS‐WT and BKS‐db/db mice (male, aged 4–5 weeks) were sourced from GemPharmatech (Nanjing, China). All mice were housed under specific pathogen‐free (SPF) environments with suitable temperature (22°C ± 2°C) and humidity (55% ± 5%), maintained on a regular light‐dark cycle, and provided with free access to food and water. Mice were monitored daily for health status and randomly assigned to experimental groups.

### 5.3 Ethical Statement

This study involving humans and animals has been approved by the Ethics Committee of the School of Stomatology, Chongqing Medical University [CQHS‐REC‐2024 (LSNo.098)], and complies with the Declaration of Helsinki. All teeth were sourced from patients at the Affiliated Stomatological Hospital of Chongqing Medical University (with informed consent obtained). Animal care and experimental protocols comply with the Animal Research: Reporting of In Vivo Experiments (ARRIVE) guidelines.

### 5.4 Ligature‐Induced Periodontitis In Vivo

Following inhalation of isoflurane for general anesthesia, sterile 5‐0 silk sutures were ligated around the cervical regions of both maxillary second molars in mice as previously reported [69]. After 2 weeks, mice were euthanized, and those that exhibited loss of ligation were excluded from the study. Gingival tissues were collected for western blotting and gene analysis, and maxillary bones were fixed in 4% paraformaldehyde for further experimentation. Depending on whether a periodontitis model was established, mice were randomly divided into the following four groups (*n* = 5), respectively: WT, WT+PD, db/db, db/db+PD.

### 5.5 Mouse Adeno‐Associated Virus Construction and Knockdown

Recombinant AAV targeting mouse *Qrich1* (NM_001114119.1) for gene knockdown was purchased from OBiO Technology. The empty vector served as a control (AAV‐Control). In 6‐week‐old male mice, expression of *Qrich1* was knocked down by injecting 2 µL of virus solution (3.5 × 10^1^ ^2^ v.g/mL) into the palatal gingival margin using a graded Hamilton syringe (33‐gauge needle). A total of three injections were administered (every other day). Two weeks post‐injection, AAV transduction efficiency was validated by qPCR and WB, followed by induction of periodontitis. To evaluate the impact of *Qrich1* knockdown on the pathogenesis of periodontitis and diabetic periodontitis, WT and db/db mice were randomized into eight experimental groups (n = 5 per group). These cohorts were established based on the administration of control vector or AAV‐*Qrich1*, as well as the induction of experimental periodontitis: WT‐Ctrl, WT‐AAV, WT‐Ctrl‐PD, WT‐AAV‐PD, db/db‐Ctrl, db/db‐AAV, db/db‐Ctrl‐PD, and db/db‐AAV‐PD.

### 5.6 Micro‐Computed Tomography Analysis

Following euthanasia, the maxillae were harvested, and then an assessment of alveolar bone resorption was conducted. Samples were scanned with a high‐resolution micro‐computed tomography system (micro‐CT; VivaCT40, SCANCO Medical AG, Switzerland) at a resolution of 12.5 µm. Three‐dimensional reconstructions were then performed using the Viva CT 40 correspondence analysis software and Mimics software (Materialise, Belgium). In the sagittal plane, the segment from the cementoenamel junction (CEJ) to the alveolar crest was assessed within the region located between the first and second maxillary molars. The regions of interest (18 × 18 pixels) extended 50 slices apically from the furcation, and BV/TV, Tb.N, Tb.Th, and Tb.Sp were analyzed with CTAn software (v1.20.3.0).

### 5.7 Histological Analysis

The mice maxillae were decalcified in 17% EDTA solution (pH = 7.4) for approximately one month. Following paraffin embedding, block slices (6 µm thick) were prepared. H&E and Masson's trichrome staining were implemented following the kit instructions (Solarbio, China). Sections were scanned with a whole‐slide scanner SLIDEVIEW VS200 (Olympus, Japan) to evaluate inflammation, alveolar bone integrity, and collagen structure.

For immunofluorescence, sections required deparaffinization and rehydration. Following antigen retrieval, sections were blocked with 10% goat serum (ZSGB‐BIO, China) at room temperature for 1 h. The primary antibody was incubated overnight at 4°C. The secondary antibody was incubated for one hour at room temperature the following day. Then, DAPI (Beyotime, China) stained the cell nucleus for 10 min. Multiplex immunofluorescence was performed according to the protocol of the PANO 5‐plex IHC Kit (Panovue, China). Following primary antibody incubation, HRP‐conjugated secondary antibodies were labeled using the tyramide signal amplification (TSA) chemistry. Antigen retrieval was repeated between each staining round to remove bound antibodies. After completing all antibody labeling steps, the cell nuclei were stained with DAPI. Primary antibodies: QRICH1 (1:100, NBP1‐82196, Novus), Caspase‐3 (1:200, 19677‐1‐AP, Proteintech), GRP78 (1:200, 11587‐1‐AP, Proteintech), NF‐κB p65 (1:200, sc‐8008, Santa Cruz), CHOP (1:100, 15204‐1‐AP, Proteintech), PERK (1:100, 24390‐1‐AP, Proteintech).

For immunohistochemistry (IHC), paraffin sections were dewaxed and rehydrated, followed by antigen retrieval using a trypsin‐based solution (ZSGB‐BIO, China) at 37°C for 30 min. After hydrogen peroxide treatment, goat serum was used to block sections to avoid non‐specific binding. Primary antibody targeting Caspase‐3 (1:100, 19677‐1‐AP, Proteintech) was incubated at 4°C overnight. Following this, the specimens were incubated with secondary antibody (HRP‐conjugated), thoroughly washed, and the signal was developed using a 3,3′‐diaminobenzidine (DAB) substrate. Staining was imaged using the fluorescent microscope Zeiss Axio Observer 7 (Zeiss, Aalen, Germany). The primary area of observation was located in the adjacent region between the first and second molars.

### 5.8 Single‐Cell RNA‐seq Data Processing and Analysis

The publicly available scRNA‐seq datasets GSE188217 and GSE280908 were retrieved from the GEO database and analyzed using the Seurat (v4.3.0) package in R (v4.3.1). After quality control to remove low‐quality cells (<200 or >6000 genes, >10% mitochondrial content), data were normalized, scaled, and subjected to PCA. Louvain clustering was performed using the top 20 principal components (resolution 0.12) and visualized via UMAP. Main cell types were characterized according to canonical markers for fibroblasts (Col1a1, Dcn), epithelial (Krt14), immune (Ptprc, Cd3d), and endothelial (Cdh5, Pecam1) cells. Fibroblasts were subset for downstream comparison between WT and db/db groups. Osteoblast subpopulations were used to compare periodontitis mice and periodontitis with T2DM mice. Differential expression was assessed using Seurat's FindMarkers (Wilcoxon rank‐sum test), and QRICH1 expression differences were visualized with FeaturePlot and VlnPlot using ggplot2 and ggpubr.

### 5.9 hPDLSCs Culture, Treatment and Osteogenic Differentiation

Premolars were extracted from 10–15 years old healthy patients (as required for orthodontic treatment). Volunteers participating in the study and their guardians signed informed consent forms. As previously described, the PDL tissues were harvested and used to culture primary hPDLSCs (passages 3–5 were used). To establish a high‐glucose inflammatory microenvironment in vitro, hPDLSCs were exposed to 10 µg/mL LPS [19] (Sigma–Aldrich, USA) and 35 mm glucose (Solarbio, China) stimulation for 48 h. 4‐phenylbutyric acid (4‐PBA) is a small‐molecule compound used to inhibit ER stress. Upon reaching 40%–50% confluence, hPDLSCs were pretreated with 5 mм 4‐PBA (Solarbio, China) for 8 h, followed by LPS and HG treatment. 5 μм BAY 11–7082 (MedChemExpress, USA) treated hPDLSCs for 24 h to inhibit NF‐κB signaling. Treatment of hPDLSCs with 0.5 µg/mL tunicamycin (TM, MedChemExpress, USA) induced ER stress. HEK293T (C5004, BDBio, China) cells were treated with 0.1 µg/mL TM.

For osteogenic induction, the osteogenic medium consisted of 50 µg/mL ascorbic acid (Sigma, USA), 10 mм β‐glycerophosphate (Sigma, USA), and 100 nm dexamethasone (Sigma, USA). Upon reaching 70%–80% confluence, hPDLSCs were cultured in an osteogenic medium. Western blot and qPCR analysis for osteogenic‐related proteins and genes were conducted 7 days after induction. After 7 days of induction, alkaline phosphatase (ALP) staining was performed using the BCIP/NBT Alkaline Phosphatase Chromogenic Assay Kit (Beyotime, China). Calcium deposition was detected using 1% Alizarin Red S Staining solution (ARS, Sigma–Aldrich, USA) 21 days post‐induction. A microscope was used for capturing images, and calcium deposition was quantified using cetylpyridinium chloride monohydrate (Solarbio, China).

### 5.10 siRNA Interference

The small interfering RNAs (siRNAs) targeting human *QRICH1* and negative control (NC) were constructed and supplied by Sangon Biotech Co., Ltd. (Shanghai, China). Prior to transfection, hPDLSCs were cultured until they reached 60% confluence, and siRNAs were transfected into hPDLSCs with Lipofectamine 3000 (Thermo Fisher Scientific, USA). In brief, transfection reagents and siRNAs were separately diluted in Gibco Opti‐MEM (Thermo Fisher Scientific, USA) serum‐reduced medium, then the diluted solutions were mixed and allowed to stand for 20 min. Subsequently, serum‐free medium was added, and the hPDLSCs were incubated for 6 h, then replaced with normal growth medium or subjected to LPS+HG or TM treatment. The interference efficiency was assessed by evaluating protein and mRNA expression after 48 h. The siRNA sequences targeting *QRICH1* are presented in Table 1.

**TABLE 1:** Design of siRNA sequences for *QRICH1* knockdown.

Primer name	sense(5′‐3′)	antisense(5′‐3′)
NC	UUCUCCGAACGUGUCACGUTT	ACGUGACACGUUCGGAGAATT
*QRICH1*‐si1	CGGAUCCUGGUGAUAAGAGAATT	UUCUCUUAUCACCAGGAUCCGTT
*QRICH1*‐si2	CCAGAACUUCUGCUUCCAAAUTT	AUUUGGAAGCAGAAGUUCUGGTT
*QRICH1*‐si3	CAACCAGUGAAGAAGCGCAAATT	UUUGCGCUUCUUCACUGGUUGTT

### 5.11 Plasmid Transfection

The overexpression plasmid targeting human QRICH1 (pCDNA3.1‑QRICH1) and the empty vector control (pCDNA3.1) were constructed and supplied by OBiO Technology Co., Ltd. (Shanghai, China). For transfection, hPDLSCs were seeded and cultured until they reached 60%–70% confluence. Transfection was performed using Lipofectamine 3000 (Thermo Fisher Scientific, USA) according to the manufacturer's instructions. Briefly, for each well of a 6‑well plate, 2.5 µg of plasmid DNA and 5 µL of P3000 reagent were diluted in 125 µL of Opti‑MEM (Thermo Fisher Scientific, USA), while 3.75 µL of Lipofectamine 3000 was diluted in another 125 µL of Opti‑MEM. The two solutions were mixed and incubated at room temperature for 15 min to form DNA‑lipid complexes. The mixture was then added to each well containing 1.75 mL of fresh complete medium. After 6–8 h of incubation, the transfection medium was replaced with normal growth medium. Following transfection, cells were treated with LPS+HG in the presence or absence of 4‑PBA or BAY 11‑7082 as indicated. Overexpression efficiency was verified by western blot and qPCR at 48 h post‑transfection.

### 5.12 RNA‐Seq Analysis

Briefly, hPDLSCs were seeded in 10 cm dishes and cultured until reaching optimal density for siRNA transfection. After 6 h of siRNA transfection, cells were treated with LPS+HG. Samples from different groups were collected 48 h later. Cells were lysed using RNAiso Plus reagent (Takara, Japan) and stored at −80°C. Sequencing and comprehensive data analysis were carried out by Novogene Biotechnology (Beijing, China) using the Illumina platform.

### 5.13 Western Blot

Periodontal ligament tissues and cultured cells were lysed using RIPA lysis (Beyotime, China) supplemented with protease and phosphatase inhibitors (Cell Signaling Technology, USA) on ice. The nucleus and cytoplasm extraction kit (Thermo Fisher Scientific, USA) was used to separate and extract cytoplasmic and nuclear proteins. Total protein concentration was determined and standardized by the BCA protein assay kit (Beyotime, China). Depending on the molecular weight of the target proteins, appropriate SDS‐PAGE gels (Beyotime, China) were used. After electrophoresis, the proteins in the gel were transferred to polyvinylidene fluoride (PVDF) membranes (Merck Millipore, USA). Then the membranes were blocked at room temperature in 5% bovine serum albumin (BSA; BioFroxx, Germany) or 5% skim milk (BioSharp, China) for 1 h, followed by incubation overnight at 4°C in primary antibody solution. The primary antibodies included: QRICH1 (1:1000, HPA037677, Sigma‐Aldrich), GRP78 (1:1000, 11587‐1‐AP, Proteintech), PERK (1:1000, 5683, CST), p‐PERK (1:1000, 3179, CST), IRE1α (1:1000, 3294, CST), eIF2α (1:1000, 5324, CST), p‐eIF2α (1:1000, 3398, CST), ATF6 (1:1000, 24169‐1‐AP, Proteintech), XBP1 (1:1000, WL00708, Wanleibio), Calnexin (1:1000, 2679, CST), CHOP (1:1000, 15204‐1‐AP, Proteintech), ATF4 (1:1000, 10835‐1‐AP, Proteintech), Caspase‐3 (1:1000, 19677‐1‐AP, Proteintech), Cleaved caspase‐3 (1:1000, 9664, CST), BAX (1:1000, 50599‐2‐Ig, Proteintech), BCL‐2 (1:1000, 12789‐1‐AP, Proteintech), RUNX2 (1:1000, 12556, CST), OPN (1:500, sc‐21742, Santa Cruz), NF‐κB p65 (1:1000, sc‐8008, Santa Cruz), p‐NF‐κB p65 (1:1000, 3033, CST), IκBα (1:1000, 10268‐1‐AP, Proteintech), p‐IκBα (1:1000, 2859, CST), β‐actin (1:5000, R380624, ZenBio), PCNA (1:5000, 10205‐2‐AP, Proteintech). The following day, the primary antibody was washed off using TBST (three times), followed by incubation with HRP‐conjugated secondary antibodies (Bio‐Rad, USA) at room temperature for 2 h. Protein bands in membranes were visualized using the ECL Kit (SQ201, Epizyme Biotech) and the ChemiDoc system (Bio‐Rad, USA). Quantitative analysis was conducted via ImageJ software (USA).

### 5.14 Quantitative Reverse Transcriptase Polymerase Chain Reaction (RT‐qPCR)

The RNAiso Plus reagent (Takara, Japan) was used to extract total RNA from periodontal tissues and cells, and reverse transcription of cDNA was conducted with the PrimeScript RT reagent kit (Takara, Japan). qPCR was employed on the CFX96 Real‐Time PCR Detection System (Bio‐Rad, USA) with SYBR Green (Takara, Japan). 18S rRNA was the internal control of reference. Relative expression levels were calculated via the 2^−ΔΔCt^ method. Primer sequences are displayed in Table 2.

**TABLE 2:** Nucleotide sequence of primers used in RT‐qPCR.

Species	Gene	Forward primer sequence (5′‐3′)	Reverse primer sequence (5′‐3′)
***Homo***	*QRICH1*	GAAGAGTACATCCGAGTAAAGGC	GCCCCTTAGAGGCCAGTGA
	*GRP78*	TGCCCATCTCTGGAAGCCTA	CCGCAGTCAAGATGCCAAAC
	*PERK*	AACTCCAGCCCAGTTCACCAA	ACACGCTCCTCTCTCTCTCCTCTA
	*IRE1α*	CATCCCCATGCCGAAGTTCA	CTGCTTCTCTCCGGTCAGGA
	*ATF6*	GACAGTACCAACGCTTATGCC	CTGGCCTTTAGTGGGTGCAG
	*CHOP*	TTCTCTGGCTTGGCTGACTG	TCCTCCTCTTCCTCCTGAGC
	*ATF4*	CCCTTCACCTTCTTACAACCTC	TGCCCAGCTCTAAACTAAAGGA
	*XBP1s*	CCCTCCAGAACATCTCCCCAT	ACATGACTGGGTCCAAGTTGT
	*IL‐6*	ACTCACCTCTTCAGAACGAATTG	CCATCTTTGGAAGGTTCAGGTTG
	*TNF ‐ α*	CCTCTCTCTAATCAGCCCTCTG	GAGGACCTGGGAGTAGATGAG
	*IL‐8*	TGGACCCCAAGGAAAACTGG	GCTTGAAGTTTCACTGGCATCT
	*IL1‐β*	GATGATGACGACCTGCTAGTGTGT	TTGGCTTATGTTCTGTCCATTGAG
	*IL‐10*	GACTTTAAGGGTTACCTGGGTTG	TCACATGCGCCTTGATGTCTG
	*RUNX2*	CTTTACTTACACCCCGCCAGTC	AGAGATATGGAGTGCTGCTGGTC
	*COL‐1*	GAGGGCCAAGACGAAGACATC	CAGATCACGTCATCGCACAAC
	*ALP*	TAAGGACATCGCCTACCAGCTC	TCTTCCAGGTGTCAACGAGGT
	*OPN*	CTCCATTGACTCGAACGACTC	CAGGTCTGCGAAACTTCTTAGAT
	*BAX*	CATGGAGCTGCAGAGGATGAT	TGCTGGCAAAGTAGAAAAGGG
	*BCL‐2*	GCTCTTCAGGGACGGGGTG	GCAGGTGCCGGTTCAGGTAC
***Mus***	*Qrich1*	ACATGCCTATCACCGTGTCCT	GGGCTAGTTATGGTCCCAGTG
	*Grp78*	GCATCACGCCGTCGTATGT	ATTCCAAGTGCGTCCGATGAG
	*Perk*	GCACTTTAGATGGACGAATCGC	TGCTGAGGCTAGATGAAACCA
	*Ire1α*	ACACTGCCTGAGACCTTGTTG	GGAGCCCGTCCTCTTGCTA
	*Chop*	ACCTTCACTACTCTTGACCCTG	GATGTGCGTGTGACCTCTGT
**Reference**	*18S*	AGTCCCTGCCCTTTGTACACA	GATCCGAGGGCCTCACTAAAC

### 5.15 Transmission Electron Microscope (TEM)

Cells were cultured in 10 cm dishes until they reached over 90% confluency. Following a phosphate‐buffered saline (PBS) wash, the cells were detached using 0.25% trypsin (HyClone, USA) and centrifuged (800 rpm, 5 min). The majority of the supernatant was aspirated, and the cell suspension was transferred to a 1.5 mL microcentrifuge tube for further centrifugation (1200 rpm, 10 min). The supernatant was completely aspirated, and the resulting cell pellet was fixed with 4% glutaraldehyde and stored at 4°C for subsequent analysis. Ultrathin sections were prepared, and the morphology of the ER was examined by a transmission electron microscope (Hitachi, Japan) under 100 kV acceleration voltage.

### 5.16 Reactive Oxygen Species (ROS) Detection

The intracellular ROS level was checked by fluorescence probe assay kit H2DCFH‐DA (Biosharp, China). Briefly, after subjecting hPDLSCs to various treatments, the cells were softly rinsed twice with PBS and then incubated in medium (serum‐free) containing 10 μм H2DCFH‐DA for 30 min under dark conditions at 37°C. Following the protocol, cells were washed twice for removing the unloaded fluorescent probe and observed under a fluorescence microscope.

### 5.17 Flow Cytometry

To assess apoptosis, hPDLSCs were treated with LPS and/or HG for 48 h. The cells were harvested, gently washed with PBS twice, and centrifuged. Then, the cells were stained with the Annexin V/propidium iodide (PI) apoptosis assay kit (Beyotime, China). The number of apoptotic cells was detected with a CytoFLEX flow cytometer (Beckman Coulter). The results were analyzed and plotted by FlowJo software (TreeStar, Ashland).

To characterize the immunophenotype of hPDLSCs, cells were washed with PBS, followed by digestion and centrifugation. Then, they were resuspended in PBS containing anti‐human stem cell surface fluorescently labeled antibodies. The marker antibodies included CD73‐PE, CD90‐FITC, CD146‐FITC, CD105‐FITC, CD45‐FITC, and CD34‐BV421 (BD Biosciences, USA). Cells not labeled with fluorophore‐conjugated antibody served as negative controls. All samples were incubated in the dark at 4°C for 1 h and detected on the BD Accuri C6 flow cytometer (BD Biosciences). Data were analyzed using FlowJo v10 software.

### 5.18 Endoplasmic Reticulum (ER) Tracker Assay

To observe morphological changes in ER, the ER‐Tracker Red kit (Beyotime, China) was employed. In short, hPDLSCs were plated and cultured, washed with PBS, and then incubated at 37°C for 30 min with pre‐warmed ER Tracker dye working solution (1:1000) to label the ER. Following incubation, the staining solution was removed, and the cells were rinsed with PBS to maintain hydration for immediate imaging. Images were captured using laser scanning confocal microscopy and fluorescence microscopy.

### 5.19 Cell Immunofluorescence (IF)

Following LPS and HG treatment, hPDLSCs were fixed with 4% paraformaldehyde for 20 min. Then, cells were treated with 0.3% Triton X‐100 (Beyotime, China) to permeabilize the cytomembrane and blocked with 5% BSA for 1 h. Next, the primary antibody QRICH1 (1:100, HPA037677, Sigma–Aldrich) was incubated with cells overnight. On the second day, they were labeled with fluorescent secondary antibody (Thermo Fisher Scientific, USA) at room temperature in the dark for 1 h, followed by nuclear staining using DAPI (Beyotime, China) for 10 min. The samples were observed under a fluorescence microscope.

### 5.20 Protein Synthesis Assay

To validate the impact of QRICH1 on protein synthesis efficiency, we performed puromycin assays. Briefly, hPDLSCs were treated with DMSO or TM, followed by 30 min incubation with puromycin (1 µm) prior to protein lysis. Afterward, the puromycin‐containing medium was removed, and the cells were washed with ice‐cold PBS. The cells were then lysed in RIPA buffer, and the supernatants were collected via centrifugation. Equal amounts of protein were loaded onto gels, subjected to electrophoresis, and subsequently transferred to the PVDF membranes. Immunoblotting was performed using anti‐puromycin antibody (1:2000, A21205, ABclonal), visualized using enhanced chemiluminescence.

### 5.21 Co‐Immunoprecipitation

Cells cultured in 10 cm dishes were lysed with a mixture of IP lysis buffer (Thermo Fisher Scientific, USA) and protease inhibitors. The protein lysates were harvested following centrifugation at 4°C (14,000 g, 10 min). For the IP procedure, 35 µL of pre‐washed protein A/G magnetic beads (MCE, USA) were conjugated with an anti‐p65 antibody at a final concentration of 10 µg/mL; mouse IgG (CST, 5415, USA) was utilized as the isotype control. The bead‐antibody complexes were resuspended and incubated at room temperature on a rotary mixer (Bio‐Rad, USA) for 1 h. Subsequently, the protein lysates (antigen samples) were incubated with the bead‐antibody complexes for 2 h with continuous agitation, followed by four rigorous wash steps. The resulting complexes were resuspended in 2× SDS loading buffer and denatured by boiling at 95°C–100°C for 5 min. The precipitated proteins were resolved via SDS‐PAGE and analyzed by western blot using primary antibodies against QRICH1 (HPA037677, Sigma‐Aldrich) and NF‐κB p65 (sc‐8008, Santa Cruz). Immunoreactive bands were visualized using an enhanced chemiluminescence (ECL) kit (Biomiky, Wuhan, China).

### 5.22 Molecular Docking and Dynamics Simulation

The target protein NF‐κB (PDB ID 1scv) was sourced from the RCSB Protein Data Bank database. Due to limited experimental structural data, the three‐dimensional structure of the QRICH1 protein was constructed using homology modeling. Prior to docking, both protein structures underwent rigorous preprocessing, including removal of all water molecules and non‐essential ligands or ions, as well as completion of missing residues and side‐chain optimization. The initial interaction conformation between NF‐κB and QRICH1 was efficiently searched using the protein‐protein rigid‐body docking software ZDOCK. The core of the ZDOCK algorithm is based on the Fast Fourier Transform (FFT), which comprehensively evaluates the binding quality of conformations, primarily considering shape complementarity, desolvation energy, and electrostatic interaction. After ZDOCK runs, the generated conformations were ranked by ZDOCK score and combined with cluster analysis to select the highest‐scoring and most representative conformation as the final complex structure for subsequent detailed analysis. The interaction interface and key residue contacts of the optimal complex were visualized and analyzed using the molecular visualization software PyMOL. Analysis focused on the distribution of residues at the binding interface, hydrogen bonds, salt bridges, and hydrophobic interactions to elucidate the specific binding mode and potential key binding sites between NF‐κB and QRICH1.

Molecular dynamics (MD) simulations in this study were performed using the GROMACS 2022 software package. The initial protein‐protein complex structure, determined through ZDOCK molecular docking, was subsequently modeled using the AMBER14sb force field. The system was positioned within a cubic simulation box, solvated using the TIP3P water model, and neutralized by adding appropriate Na^+^ or Cl^−^ counterions. Periodic boundary conditions (pbc = xyz) were applied throughout this simulation. The system first underwent Energy Minimization (EM) using the Steepest Descent algorithm. This was followed by a two‐stage equilibration: 500 ps in the canonical (NVT) ensemble and 500 ps in the isothermal‐isobaric (NPT) ensemble. During equilibration, temperature was maintained at 300 K using the V‐rescale thermostat (sτt = 1.0 ps), and pressure in the NPT stage was maintained at 1.0 bar using the Parrinello‐Rahman barostat (τp = 10.0 ps) with isotropic coupling. Following equilibration, a 100 ns (50,000,000 steps) production MD run was performed using the Verlet integrator with a time step (dt) of 2 fs (0.002 ps). All hydrogen bonds were constrained using the LINCS algorithm (lincs‐order = 6). The non‐bonded interactions were treated with the Verlet cutoff scheme, using a cutoff distance of 1.2 nm (rlist = rvdw = 1.2 nm) for both short‐range van der Waals (VDW) and electrostatic interactions. VDW forces were smoothed using the Force‐switch modifier between 1.0 nm and 1.2 nm, and a Dispersion Correction (DispCorr = EnerPres) was applied to the energy and pressure. Electrostatic interactions were calculated using the Particle Mesh Ewald (PME) method (rcoulomb = 1.2 nm, fourierspacing = 0.16 nm). Trajectory (xtc format) and energy (edr format) data were saved every 10 ps (5000 steps).

### 5.23 Cell Counting Kit‐8

The hPDLSCs were seeded into 96‐well culture plates. After adhesion, they were treated with complete medium containing varying doses of BAY 11–7082 (0–5 µm) to assess the impact on the proliferative ability of hPDLSCs. Next, at the specified time intervals, cells were incubated with 10 µL CCK‐8 reagent (Dojindo, Japan) in 100 µL fresh medium at 37°C for 3–4 h. Then the absorbance was measured at 450 nm by a Spectramax iD5 microplate reader (Molecular Devices). Each group contained four parallel replicates.

### 5.24 Statistical Analysis

All data were analyzed using GraphPad Prism software (version 10.1.2, San Diego, USA). Results were presented as the mean ± standard error of the mean (SEM). For multiple comparisons, one‐way analysis of variance (ANOVA) was performed, followed by Tukey's multiple comparison test. Statistical significance was defined as p < 0.05; significance levels were shown by asterisks in the figures. All experiments were independently repeated at least three times, with representative data shown.

## Author Contributions

**Han Li**: conceptualization, methodology, investigation, data curation, validation, writing – original draft, writing – review and editing. **Xiaoyu Yang**: methodology, investigation, data curation, visualization. **He Wang**: methodology, investigation. **Yunchun Kuang**: investigation. **Yiyao Hu**: software. **Houxuan Li**: methodology. **Shuhong Li**: data curation. **Deping Zeng**: resources. **Jie Li**: supervision, funding acquisition, writing – reviewing and editing, project administration. **Jinlin Song**: funding acquisition, supervision, project administration.

## Conflicts of Interest

The authors declare no conflicts of interest.

## Supporting information




**Supporting File**: advs76650‐sup‐0001‐SuppMat.docx.

## Data Availability

The data that support the findings of this study are available from the corresponding author upon reasonable request.
