# Supplementary material for: QRICH1 Disrupts Endoplasmic Reticulum Homeostasis and Amplifies NF‐κB Signaling in Periodontal Ligament Stem Cells to Exacerbate Diabetic Periodontitis
Source: Adv Sci (Weinh). 2026 Jul 20:e76650. Online ahead of print. doi: 10.1002/advs.76650 (PMC13383152; doi:10.1002/advs.76650)
Supplement: Supplementary file 1 — Supporting File: advs76650‐sup‐0001‐SuppMat.docx. [file ADVS-9999-e76650-s001.docx]

Supporting Information

QRICH1 disrupts endoplasmic reticulum homeostasis and amplifies NF-κB signaling in periodontal ligament stem cells to exacerbate diabetic periodontitis

*Han Li, Xiaoyu Yang, He Wang, Yunchun Kuang, Yiyao Hu, Houxuan Li, Shuhong Li, Deping Zeng, Jie Li* ^*^*, Jinlin Song* ^*^

Figure S1


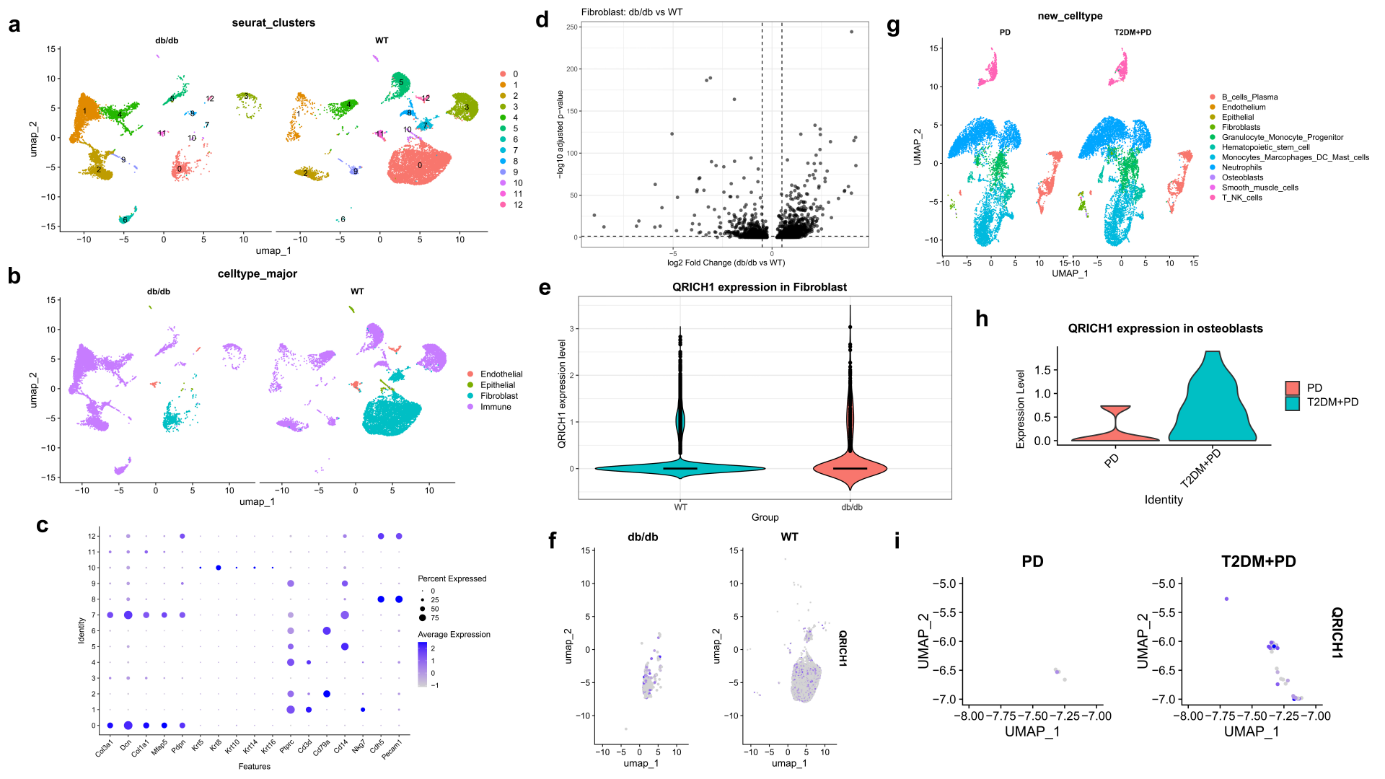


**Figure S1. Analyses of scRNA-seq datasets to assess the expression of QRICH1.**

a) Uniform manifold approximation and projection (UMAP) visualization of cells from WT and db/db mouse periodontal tissues, identifying 13 transcriptionally distinct clusters. b) Classification of the 13 clusters into four major cell types (fibroblasts, epithelial cells, immune cells, and endothelial cells) based on canonical lineage markers. c) Representative marker genes for the four major cell types. d) Differential expression analysis revealed significant transcriptional differences in fibroblasts between WT and db/db mice. e, f) Violin plot (e) and UMAP feature visualization (f) revealed significantly increased expression of QRICH1 in fibroblasts from db/db mice compared to WT (Wilcoxon rank-sum test, ***P* < 0.01). g) UMAP visualization displays cell populations in periodontitis mice (with or without T2DM), annotated as 11 distinct cell types based on classical markers. h, i) Violin plot (h) and UMAP feature visualization (i) revealed an upward trend in QRICH1 expression in osteoblasts from T2DM+PD mice compared to the PD group.

Figure S2


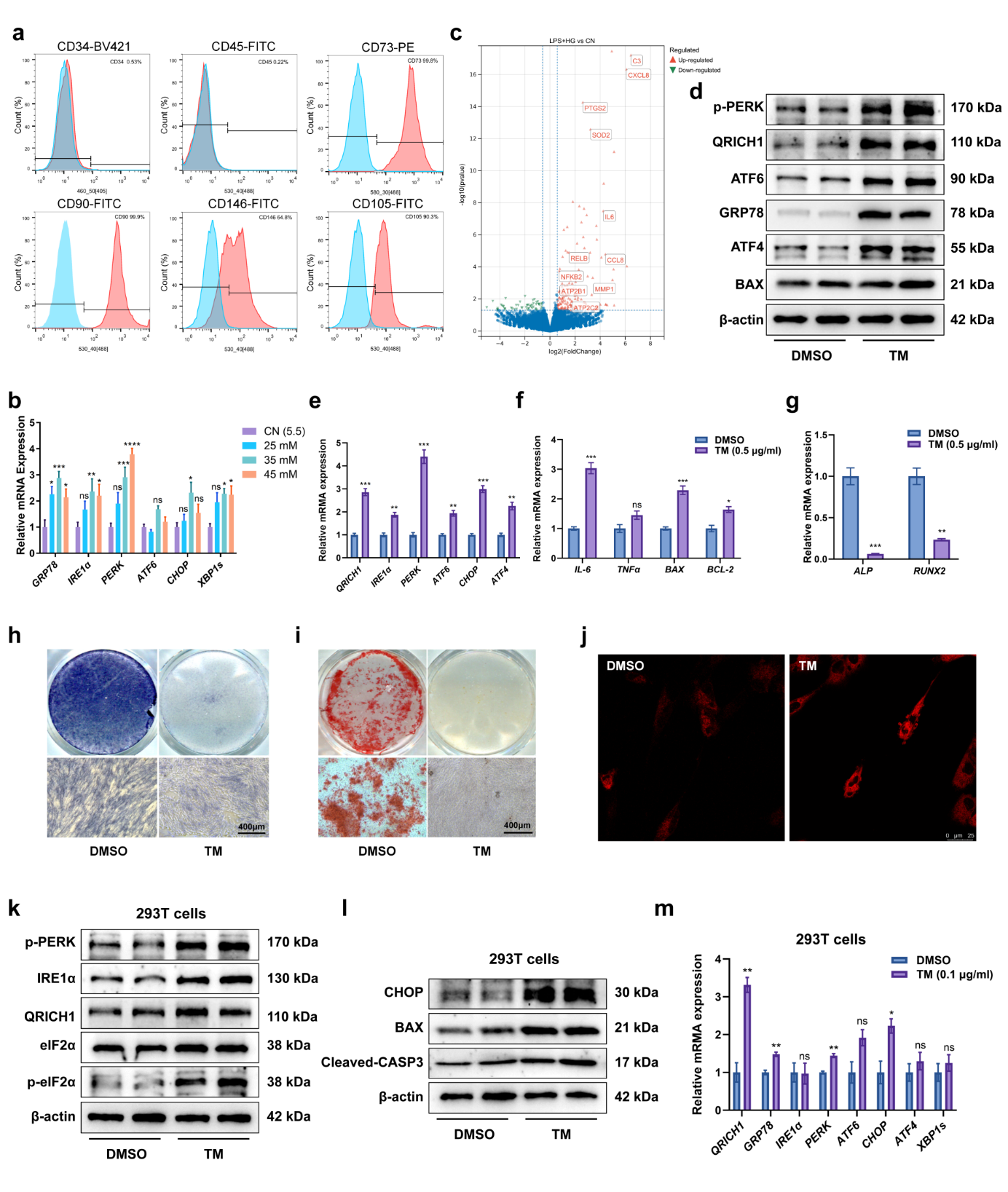


**Figure S2. Activation of ER stress by LPS+HG or TM treatment impairs hPDLSC function.**

a) Flow cytometric analysis of stem cell surface markers revealed that hPDLSCs were positive for CD73, CD90, CD146, and CD105 but negative for CD34 and CD45 (n = 3 per group). b) Relative mRNA expression levels of ER stress-related genes in hPDLSCs treated with different glucose concentrations for 24 hours; 35 mM was selected for subsequent HG experiments (n=3 per group). c) Volcano plot of differentially expressed genes (DEGs) between hPDLSCs in CN and LPS+HG groups, highlighting changes in markers related to inflammatory response, NF-κB signaling pathway, and cellular calcium homeostasis. d) Western blot analysis of QRICH1, ER stress-related proteins, and apoptosis protein BAX in hPDLSCs with or without TM stimulation (n = 3 per group). e, f) Relative mRNA expression levels of ER stress-related genes (e) and inflammatory and apoptosis markers (f) in hPDLSCs treated with TM or DMSO for 24 hours (n=3 per group). g-i) qPCR analysis revealed that osteogenesis-related genes *ALP* and *RUNX2* were significantly downregulated in hPDLSCs following TM treatment (g); ALP (h) and ARS (i) staining further validated that TM-induced ER stress impaired the osteogenic potential of hPDLSCs. (n=3 per group). Scale bars, 400 µm. j) ER-Tracker Red fluorescence labeled the ER in hPDLSCs treated with TM. Scale bars, 25 μm. k, l) Western blot analysis of QRICH1, ER stress-related proteins, and apoptosis proteins in 293T cells with or without TM stimulation (n = 3 per group). m) qPCR analysis of *QRICH1* and ER stress-related genes in 293T cells with or without TM stimulation (n = 3 per group). CN: controls; LPS: lipopolysaccharide; HG: high glucose; DMSO: dimethylsulfoxide; TM: tunicamycin. Bar graphs: Values are presented as mean ± SEM. ns, not significant. **P* < 0.05, ***P* < 0.01, ****P* < 0.001, *****P* < 0.0001.

Figure S3


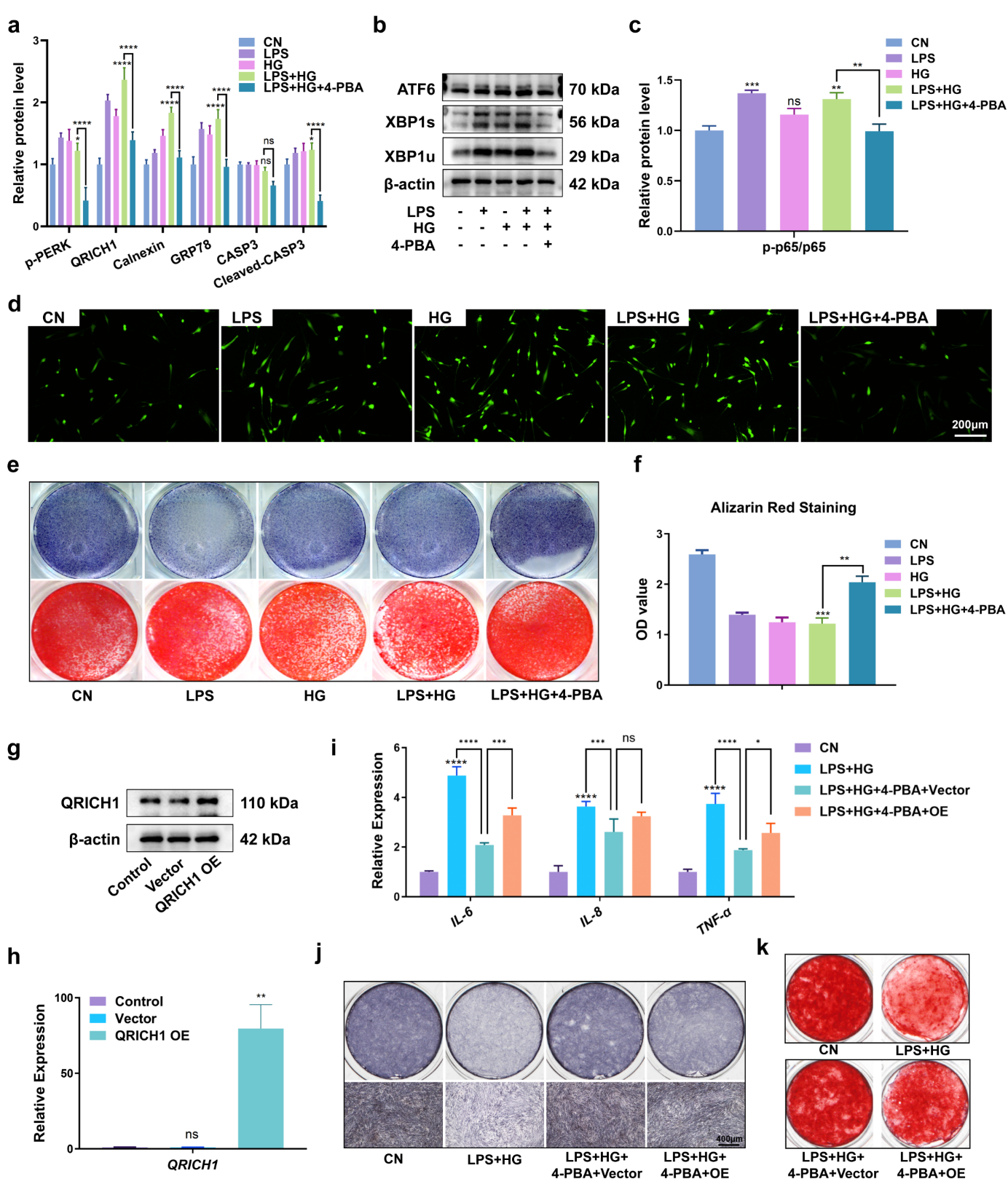


**Figure S3. Validation of UPR branch inhibition by 4-PBA and effects of QRICH1 overexpression on cellular functions.**

a) Quantification of p-PERK, QRICH1, Calnexin, GRP78 and cleaved caspase-3 protein levels using ImageJ software (n=3 per group). b) Western blot analysis of ATF6, XBP1u and XBP1s expression in hPDLSCs with 4-PBA treatment. c) Quantification of p-p65/p65 expression following 4-PBA treatment (n=3 per group). d) Detection of intracellular ROS levels in hPDLSCs following 4-PBA treatment. Scale bars, 200 μm. e,f) ALP and ARS staining demonstrated the restoration of osteogenic activity in hPDLSCs following 4-PBA treatment (n = 3 per group). g,h) Western blot and qPCR analysis confirming efficient QRICH1 overexpression in hPDLSCs (n=3 per group). i) qPCR analysis showed that QRICH1 overexpression elevated IL-6 and TNF-α mRNA levels (n = 3 per group). j,k) ALP and ARS staining indicated that QRICH1 overexpression reversed the protective effect of 4-PBA on osteogenesis in hPDLSCs. Scale bars, 400 μm. CN: controls; LPS: lipopolysaccharide; HG: high glucose; 4-PBA: 4-phenylbutyrate; CASP3: caspase-3; ALP: alkaline phosphatase; ARS: Alizarin Red S; OE: overexpression. Bar graphs: Values are presented as mean ± SEM. ns, not significant. **P* < 0.05, ***P* < 0.01, ****P* < 0.001, *****P* < 0.0001.

Figure S4


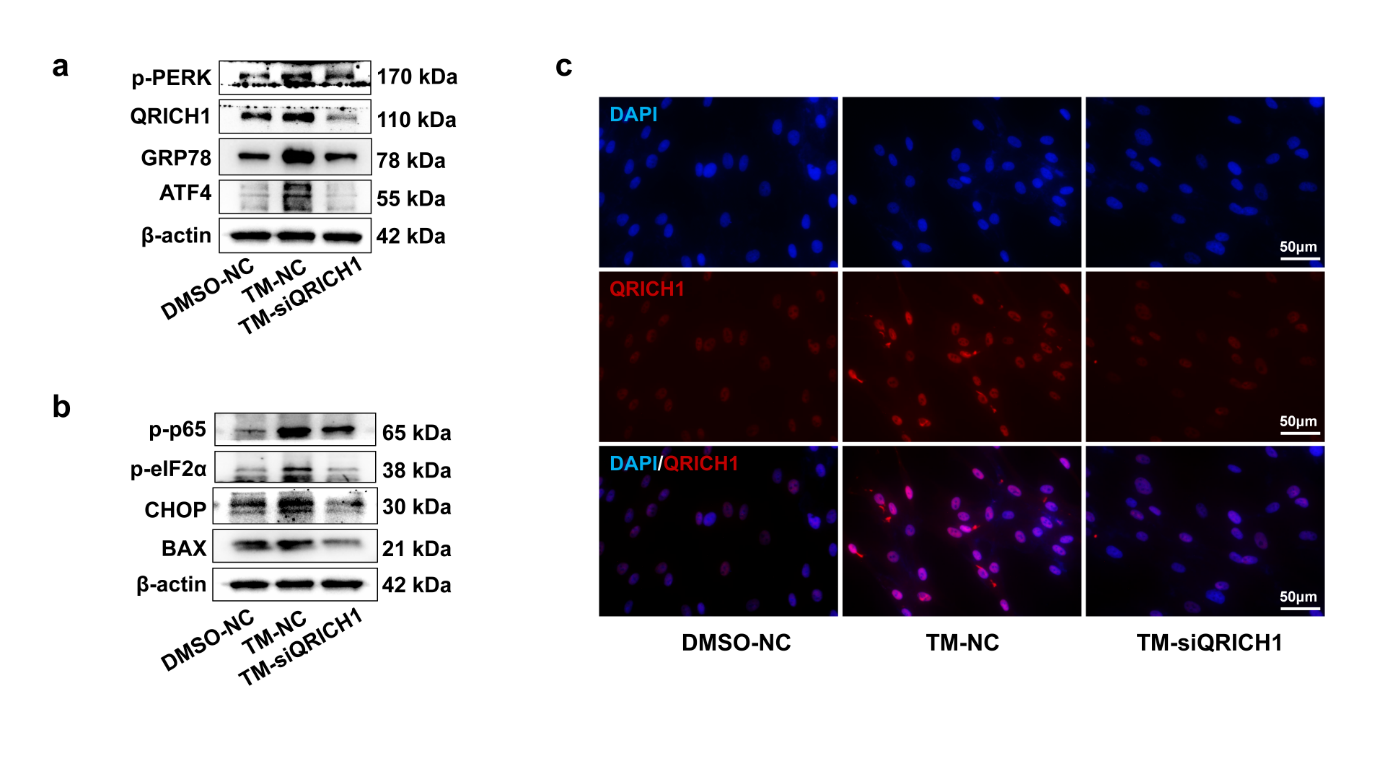


**Figure S4. TM stimulation validated the impact of *QRICH1* knockdown on the UPR signaling pathway.**

a, b) Following TM stimulation, hPDLSCs were treated with si-*QRICH1*, and the levels of QRICH1, ER stress markers (p-PERK, GRP78, ATF4, p-eIF2α), p-p65, and apoptotic markers (CHOP, BAX) were detected by western blot analysis (n = 3 per group). c) Immunofluorescence staining revealed the expression and localization of QRICH1 in hPDLSCs from different groups. Scale bars, 50 μm. DMSO: dimethylsulfoxide; NC: negative control; TM: tunicamycin.

Figure S5


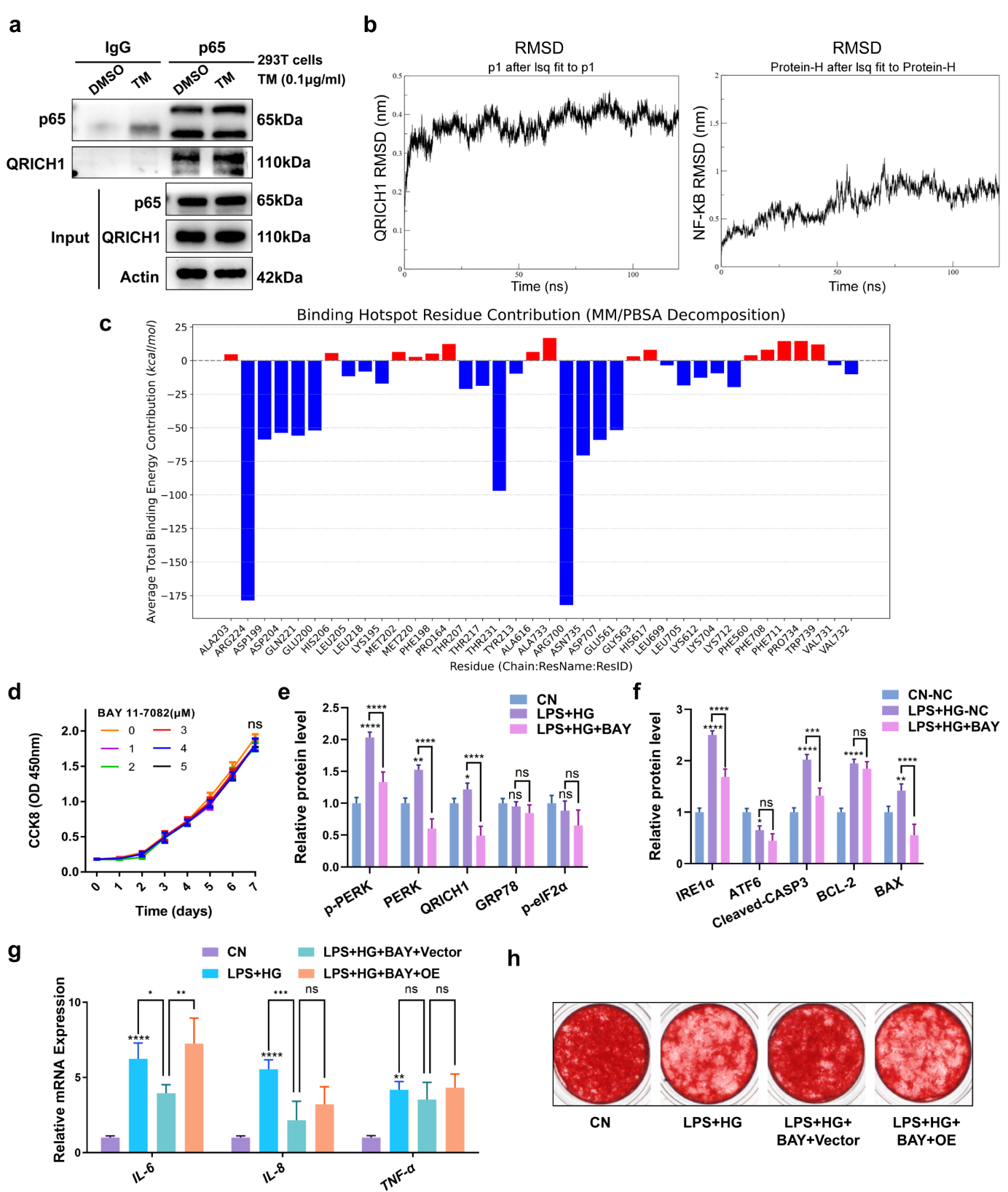


**Figure S5.** **Additional validation of the QRICH1-NF-κB interaction and feedback loop.**

a) Co-IP analysis showed the interaction between p65 and QRICH1 in 293T cells under TM treatment. b) RMSD analysis from molecular dynamics simulation revealed greater conformational flexibility of NF-κB compared to QRICH1. c) Energetic analysis identified ASP-204 of QRICH1 and ASP-707 of NF-κB as the key residues driving the interaction. d) CCK-8 viability assay screened the concentration of BAY 11-7082 for hPDLSCs treatment (5 μM for subsequent experiments) (n=3 per group).e,f) Quantification with ImageJ software showed that BAY 11-7082 treatment markedly reduced the expression of QRICH1, PERK, IRE1α, the apoptotic proteins cleaved caspase-3 and BAX in hPDLSCs (n=3 per group). g) qPCR analysis demonstrated that QRICH1 overexpression increased *IL-6* mRNA levels under BAY 11-7082 treatment (n = 3 per group). h) ARS staining showed that QRICH1 overexpression counteracts the osteogenic rescue effect of NF-κB inhibition. CN: controls; LPS: lipopolysaccharide; HG: high glucose; BAY: BAY 11-7082; OE: overexpression; RMSD: root-mean-square deviation; ARS, Alizarin Red S. Bar graphs: Values are presented as mean ± SEM. ns, not significant. **P* < 0.05, ***P* < 0.01, ****P* < 0.001, *****P* < 0.0001.

Figure S6


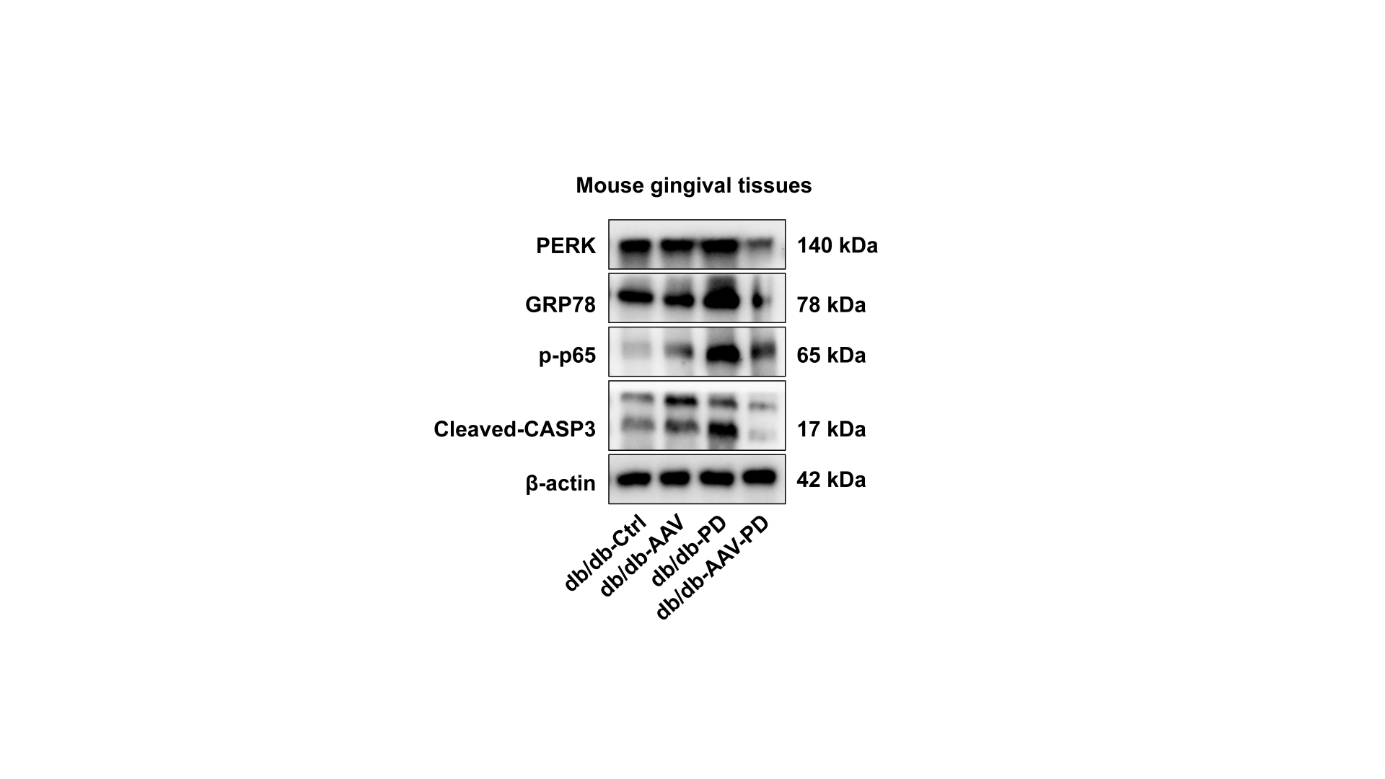


**Figure S6. Western blot validation of AAV-mediated *Qrich1* knockdown *in vivo*.**

Western blot analysis of PERK, GRP78, cleaved caspase-3, and p-p65 in gingival tissues from db/db mice treated with AAV-Control or AAV-*Qrich1*. db/db: diabetic mice; PD: periodontitis; AAV: adeno-associated virus.
